# Gendered Paths Into STEM-Related and Language-Related Careers: Girls’ and Boys’ Motivational Beliefs and Career Plans in Math and Language Arts

**DOI:** 10.3389/fpsyg.2019.01243

**Published:** 2019-06-06

**Authors:** Rebecca Lazarides, Fani Lauermann

**Affiliations:** ^1^Department of Education, Universität Potsdam, Potsdam, Germany; ^2^Center for Research on Education and School Development (IFS), TU Dortmund University, Dortmund, Germany

**Keywords:** gendered motivational beliefs, career plans, mathematics, language arts, dimensional comparison

## Abstract

Women are often underrepresented in math-intensive fields like the physical sciences, technology, engineering and mathematics. By comparison, boys relative to girls are less likely to strive for jobs in social and human-services domains. Relatively few studies have considered that intra-individual comparisons across domains may contribute to gendered occupational choices. This study examines whether girls’ and boys’ motivational beliefs in mathematics and language arts are predictive of their career plans in these fields. The study focusses on same domain and cross-domain effects and investigates bidirectional relations between motivational beliefs and career plans. Data for this study stem from 1,117 ninth and tenth graders (53.2% girls) from secondary schools in Berlin, Germany. Findings show systematic gender differences in same-domain effects in mathematics: girls’ comparatively lower mathematics self-concept and intrinsic value predicted a lower likelihood of striving for a math-related career. Cross-domain effects were not related to gender-specific career plans, with only one exception. Girls’ lower levels of intrinsic value in mathematics corresponded to a higher likelihood of striving for a career in language-related fields, which subsequently predicted lower levels of intrinsic value in mathematics. This finding points to a need to address both gender-specific motivational beliefs and gender-specific career plans in school when aiming to enhance more gender equality in girls’ and boys’ occupational choices.

## Introduction

A substantial amount of research has focused on social and individual factors contributing to persistent gender disparities in the selection and pursuit of particular career paths (for an overview, see for example Watt, 2016; Wang and Degol, 2017). This research shows that women are often underrepresented in math-intensive fields like science, technology, engineering and mathematics (STEM) (Watt et al., 2012; Watt, 2016). By comparison, boys relative to girls are less likely to strive for jobs in social and human-services domains (Su and Rounds, 2015; Wolter et al., 2015), which often require higher levels of verbal than math skills (see National Center for O^∗^Net Development, 2014; Lauermann et al., 2015). Research based in expectancy-value theory (EVT; Eccles et al., 1983) and the dimensional comparison theory (DCT; Möller and Marsh, 2013) suggests that systematic differences in students’ domain-specific motivational beliefs (i.e., academic self-concepts and task values) can contribute to such gender-specific career paths. Girls often report lower levels of intrinsic and utility value of mathematics than boys (Gaspard et al., 2015) as well as lower self-concept of ability in this domain (Marsh and Yeung, 1998). Boys in turn report lower levels of intrinsic value and self-concept in language arts (Jacobs et al., 2002; Watt, 2004). Differences in academic beliefs about mathematics and language arts can thus potentially shape subsequent career preferences for occupations that are perceived as either math-intensive (e.g., STEM) or verbal-intensive (e.g., communication, teaching). Relatively few studies, however, have considered that intraindividual comparisons across such domains as math and language arts may also contribute to gendered educational and occupational choices (Nagy et al., 2006; Lauermann et al., 2015). A choice against a math-intensive career, for instance, may be linked to a comparatively higher interest in the verbal domain rather than a low interest in math.

In the present study, we build upon this research and examine whether adolescent girls’ and boys’ motivational beliefs in mathematics and language arts are predictive of their career plans in these fields. In line with previous research (Lauermann et al., 2017; Lazarides et al., 2017), we understand career plans both as an outcome and as a precursor of students’ motivational beliefs. Academic motivations in math and language arts may lead students to choose careers for which these domains are important; at the same time, choosing a career that requires relatively high levels of math or verbal skills may increase students’ motivations to engage in these academic domains as a means of accomplishing their career goals (for the math domain, see e.g., Lauermann et al., 2017; Lazarides et al., 2017). Therefore, we investigate potential bidirectional relations between students’ motivational beliefs and career plans.

### Gendered Motivational Beliefs and Career Plans

Eccles and colleagues’ expectancy-value theory (EVT; Eccles et al., 1983, 1998) proposes that individuals’ motivational beliefs – defined as their subjective valuing of and expected success in a given task – are important predictors of students’ task-related activities, achievements, career plans, and career attainment. Task values are defined as “the quality of the task that contributes to the increasing or decreasing probability that an individual will select it” (Eccles, 2005, p. 109) and are described in terms of four components: students’ task-related enjoyment (intrinsic value), the perceived usefulness of activities and tasks for own short- and long-term goals (utility value), the personal importance of doing well on a given task (attainment value), and the subjective cost related to engaging in given activities and tasks (cost value). In this study, we focus on students’ intrinsic and utility values because these components have been shown to be important antecedents of students’ educational and occupational choices (Nagy et al., 2006; Watt et al., 2012; Lauermann et al., 2017; Lazarides et al., 2019). Success expectancies are defined as individuals’ beliefs about how well they will do on upcoming tasks, either in the immediate or long-term future (Eccles and Wigfield, 2002). The key conceptual difference between students’ success expectancies and academic self-concept of ability is that success expectancies refer to future achievements (Wigfield and Eccles, 2000), whereas academic self-concept refers to past accomplishments that inform students’ self-evaluations (Marsh et al., 2018). However, these two constructs are often not empirically distinguishable (Wigfield and Eccles, 2000), possibly because students use their past experiences as an important reference point to estimate the subjective likelihood of succeeding in a given academic domain in the future (e.g., Marsh et al., 2005). When both constructs reference the same domain (e.g., math or reading), they typically form one factor (e.g., Eccles and Wigfield, 1995; Wigfield and Eccles, 2000). Similar to previous research in EVT, we focus on students’ academic self-concept of ability as an important antecedent of students’ expected success in a given domain. Studies have shown that students’ academic self-concept is highly related to their achievement (Marsh et al., 2005), whereas their task values are comparatively more strongly related to career choices and aspirations (e.g., Meece et al., 1990; Watt et al., 2012; Lazarides and Watt, 2015; Lauermann et al., 2017).

A number of studies demonstrate persistent gender differences in adolescents’ domain-specific task values (e.g., Watt et al., 2012; Gaspard et al., 2015) and academic self-concept of ability (Marsh and Yeung, 1998). Girls, compared to boys, tend to report lower levels of intrinsic value (Frenzel et al., 2010; Watt et al., 2012; Gaspard et al., 2015) and lower academic self-concepts in mathematics (Marsh and Yeung, 1998). Girls also report lower levels of perceived utility of mathematics for their future life and for their job prospects (Gaspard et al., 2015). By comparison, boys report lower self-concept of ability (Marsh and Yeung, 1998; Arens and Jansen, 2016) and lower levels of interest in language-related domains (Yeung et al., 2011). Such gender-specific motivational beliefs are associated with gender differences in students’ educational and career paths (Watt et al., 2012; Lauermann et al., 2017). In the math domain, girls tend to report comparatively lower levels of motivation and lower levels of interest in math-intensive careers. In an Australian sample of adolescents, Watt et al. (2012) found that girls participated less often in math courses than did boys and less often aspired to math-related careers. In a longitudinal U.S. sample, Lauermann et al. (2017) found a weak positive association between gender and grade 12 self-concept of ability in mathematics favoring male students, and male students were more likely to strive for and attain math-related careers as adults. In a longitudinal sample in Germany, Lazarides et al. (2017) found that boys reported higher levels of interest and utility value in math and were more likely than girls to strive for math-related careers. Regarding gender differences in domains in which women are typically overrepresented, Nagy et al. (2006) found that boys were less likely than girls to choose an advanced biology course in grade 12, and findings reported in Lauermann et al. (2015) suggest that girls were more likely than boys to consider human services occupations, which tend to be verbal-intensive. Building on this previous evidence, we examine whether gender differences in students’ academic motivations, namely self-concepts of ability and task values, are linked to corresponding differences in adolescents’ career choices. We focused on the domains of mathematics and language arts due to their critical role for a variety of occupational fields and due to persistent gender differences in these domains.

### Dimensional Comparisons and Gendered Career Plans

Individuals’ motivational beliefs are influenced by internal and external comparison processes (Eccles, 2009; Möller and Marsh, 2013). Individuals tend to assess their own skills by comparing their performance in a given domain with the performance of relevant peers (*external comparisons*) and by comparing their levels of performance across different domains (*internal comparisons*). Such cross-domain comparison processes play a central role in the development of students’ academic self-concept of ability, as described in the internal/external frame of reference model (I/E model; Marsh, 1986). According to the I/E model, a continuum of core academic self-concepts exists, which include students’ self-concept in the verbal domain and their self-concept in the math domain (Marsh et al., 2015). Students evaluate their abilities by comparing their performance in a given domain to their own past performances in this domain, to the observed performance of relevant peers, or to their own performance across domains. Consistent with the theoretical assumptions of the I/E model, a number of studies have documented negative contrast effects across the math and verbal domains (e.g., Brunner et al., 2008; Möller et al., 2009, 2011; Niepel et al., 2014). Whereas students’ verbal achievement positively predicts their verbal self-concept of ability (“*same-domain effect*”), it has a negative effect on students’ self-concept of ability in math (“*cross-domain effect*”). High performance in the verbal domain sets a high standard against which students’ math performance is being compared, which then negatively affects their self-evaluated competence in math. Analogous contrast effects have been documented with regard to students’ math performance and verbal self-concept of ability.

The dimensional comparison theory (DCT; Möller and Marsh, 2013) was developed as an extension of the I/E model (Marsh et al., 2015). A central contribution of DCT (Möller and Marsh, 2013) is that it incorporates contrast effects, assimilation effects, and same-domain effects across a wide range of academic subjects that are relatively similar (“near comparisons”) or dissimilar (“far comparisons”). Negative contrast effects, or cross-domain effects, of students’ achievement on their self-concept of ability are likely to apply across dissimilar domains like math and language arts (e.g., a negative effect of math achievement on verbal self-concept of ability and vice versa); positive assimilation effects are likely to apply across subjects that are similar to each other (e.g., a positive effect of math achievement on physics self-concept of ability); and same-domain effects apply within the same domain (e.g., a positive effect of math achievement on math self-concept of ability).

Furthermore, DCT expands upon the IE-framework by focusing on the “why,” “with what” and “with what effect” questions of dimensional comparisons (Möller and Marsh, 2013). Notably, Möller and Marsh (2013, p. 553) point out that the vast majority of available evidence on the effects of dimensional comparisons (i.e., the “with what” question) has focused on students’ domain-specific academic self-concepts, even though dimensional comparisons can also affect other outcomes such as mood, course selection, or career choices. Dickhäuser et al. (2005), for example, focused on academic self-concept and course selection in biology and chemistry, and showed significant negative paths from students’ self-concepts on the selection of non-corresponding subjects. Lauermann et al. (2015) examined the relations between adolescents’ motivational beliefs across two academic domains, English and math, on their math/science-related and human services-related career plans and identified significant negative paths from students’ English self-concept and English task values on their career plans in math.

In the present study, we focus on dimensional comparison effects among motivational beliefs (academic self-concept and task values) and career plans in math and language-related domains and examine whether these dimensional comparison effects may contribute to gender disparities in adolescents’ domain-specific motivations and career plans.

A few recent studies have examined gender differences in educational (Nagy et al., 2006; Wang et al., 2013; Guo et al., 2017) and occupational choices (Parker et al., 2012; Lauermann et al., 2015) based on the theoretical assumptions of EVT and DCT. These studies showed that dimensional comparison effects might partially explain gender-specific educational and occupational choices. For instance, in a study in the United States, girls reported significantly higher valuing of English as a subject domain than did boys, which not only positively predicted their preference for human-services careers but also negatively predicted their interest in pursuing careers in math and science (Lauermann et al., 2015). Wang et al. (2013) showed similar effects for STEM careers in a U.S. sample; girls were more likely than boys to have high math and high verbal ability, which corresponded to a lower likelihood of pursuing STEM careers. Another study with German adolescents (Nagy et al., 2006) found that having high levels of math achievement and math self-concept of ability negatively predicted boys’ enrolment in advanced biology courses, but did not affect girls’ enrolment in such courses. These studies thus suggest that negative cross-domain effects may differentially affect girls’ and boys’ educational and career choices.

Taken together, this evidence suggests that dimensional comparison processes can contribute to gendered educational and occupational choices. However, these studies have focused on ability (Wang et al., 2013), single task value components such as intrinsic value (Nagy et al., 2006), or on a composite score of all task values (Lauermann et al., 2015). Thus, the role of different motivational components like students’ intrinsic, utility, and attainment value has not been systematically examined. Furthermore, the reciprocal longitudinal associations between students’ academic motivations and career plans remain understudied (e.g., Lauermann et al., 2017). Finally, most of the cited research has focused on the math domain, and only a handful of studies have focused on career plans in verbal domains (e.g., Durik et al., 2006). Thus, the present study examines the longitudinal relations between girls’ and boys’ task value components (intrinsic, utility, and attainment value), academic self-concepts, and career plans in mathematics and language-related domains.

### The Present Study

Informed by both EVT and DCT, the primary objective of this longitudinal study is to examine the predictive effects of student gender on their motivational beliefs and career plans in mathematics and language arts. We examine same-domain and cross-domain effects and consider the potential reciprocity of the relations between motivational beliefs and career plans. Based on our review of literature and theoretical considerations, we derived a set of five hypotheses focusing on gender differences, same-domain associations, and cross-domain effects in the math and language arts domains. First, we hypothesize that girls will report lower motivational beliefs (academic self-concept and task values) in mathematics than boys, and that girls will be less likely than boys to strive for careers in math-intensive fields (*Hypothesis 1*). We also hypothesize that boys will report lower motivational beliefs (academic self-concept and task values) in language arts than girls, and that boys will be less likely than girls to strive for careers in language-related fields (*Hypothesis 2*). Third, we expect to find positive same-domain associations between motivational beliefs and career plans, such that mathematics (vs. language-related) task values and self-concepts will positively predict math-related (vs. language-related) career plans (*Hypothesis 3*). We also expect to find negative cross-domain effects between math- and language arts-related motivational beliefs and career plans; we expect that mathematics (vs. language-related) task values and self-concepts will negatively predict language-related (vs. math-related) career plans and vice versa (*Hypothesis 4*). Additionally, in line with the I/E model (Marsh, 1986), we expect to find positive same-domain effects, and negative cross-domain effects among students’ grades and their motivational beliefs (self-concept of ability and task values) (Gaspard et al., 2018) (*Hypothesis 5*). Lastly, we expect to identify gender-specific (same-domain and cross-domain) motivational processes. We assume that the predictive effects of students’ gender on career plans in math- and language-related domains are at least partly attributable to gender differences in motivational beliefs in math and language arts (*Hypothesis 6*).

**FIGURE 1:**
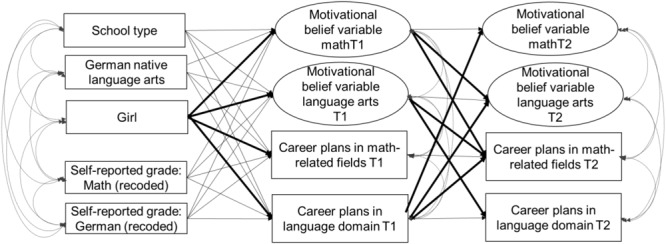
Schematic model of the tested relations.

The following control variables were included in all analyses: whether German was a native language and school type (academic track vs. comprehensive school). The schematic model of the tested relations is depicted in Figure 1.

## Materials and Methods

### Sample

Data was drawn from the German Move Study (Motivation and Valuing in Mathematics; Lazarides and Rubach, unpublished), which examines the relations between students’ perceptions of their mathematics teachers’ beliefs, perceptions of teachers’ instructional behaviors, and students’ motivations. In the longitudinal study Move, data was obtained from parents, students, and their mathematics teachers concerning perceived teaching quality, learning support and motivation for mathematics at three measurement points, two of which were included in the present study^.^ The participating schools were randomly selected from a list of all secondary schools in Berlin, and data were collected by trained research assistants at the end of a compulsory class, approximately 2 months after the beginning of the 2015 school year (Time 1), as well as after the mid- year mark in the spring of 2016 (Time 2). The survey administration took approximately 30 min. In this study, we used the data from 1,117 students (age: *M* = 14.59 years, *SD* = 0.88) who participated at the first time point. A total of 746 9th (54.0%) and 10th graders (46%) (age: *M* = 14.50 years, *SD* = 0.86) participated at the first two time points included in this study. Written informed consent was obtained from the parents of the participants. The Berlin Senate for Education, Youth, and Research approved the study. An ethics approval was not required at the time the study was conducted as per the then applicable institutional and national guidelines and regulations. The students (53.2% girls) came from 58 classrooms across 13 secondary schools in Berlin, Germany. The sample consisted of ninth (48%) and tenth (52%) graders. Most students (69.8%) reported that they were native speakers of German. Approximately half of the students attended a gymnasium school (the highest academic track in Germany; 51.8%), whereas the remaining students attended comprehensive schools (a type of secondary school that provides courses for different ability levels; 48.2%). Students’ participation was voluntary.

### Measures

The following sections provide an overview of all scales used in this study (the items are reported in Appendix A).

#### Ability Self-Concept

Students’ self-concepts in mathematics and in language arts were assessed with an 8-item scale, with answer options ranging from 1 to 5 (see Steinmayr and Spinath, 2010). Four parallel domain-specific items were used to assess student’s self-concept in mathematics (e.g., “I think I am … in mathematics” from “1 [not talented] to 5 [very talented]”) and language arts (e.g., “I think I am … in German” from “1 [not talented] to 5 [very talented]”). The scales had very good internal consistency for math (α = 0.87 at Time 1 and α = 0.88 at Time 2) and language arts (α = 0.86 at Time 1 and α = 0.87 at Time 2).

#### Utility Value

Students’ utility values in mathematics and language arts were assessed with a six-item scale based on Steinmayr and Spinath (2010), with answer choices ranging from 1 (*does not apply at all*) to 5 (*fully applies*). Three parallel items were used to assess utility value in mathematics (e.g., “Mathematics is useful for my future.”) and language arts (e.g., “German is useful for my future”). The internal consistencies of these scales were very good in math (α = 0.88 at Time 1 and α = 0.89 at Time 2) and language arts (α = 0.91 at Time 1 and α = 0.91 at Time 2).

#### Intrinsic Value

Students’ intrinsic values in mathematics and language arts were assessed with a six-item scale based on Steinmayr and Spinath (2010), with answer choices ranging from 1 (*does not apply at all*) to 5 (*fully applies*). Similar to utility value, three parallel items were used to assess intrinsic value in mathematics (e.g., “I like mathematics”) and language arts (e.g., “I like German). The internal consistencies were very good in math (α = 0.92 at Time 1 and α = 0.92 at Time 2) and language arts (α = 0.93 at Time 1 and α = 0.92 at Time 2).

#### Career Plans

Students’ mathematics-related career plans were assessed with the item “What occupation do you think are you going to have when you are 30 years old?” Two independent coders coded the math-relatedness of students’ open-ended answers for relatedness to mathematics and language domains per nominated career using the Occupational Information Network (O^∗^NET; National Center for O^∗^Net Development, 2014) to quantify the importance of “knowledge of arithmetic, algebra, geometry, calculus, statistics, and their applications” (for level of importance of mathematics for the job) and of “the structure and content of the English language including the meaning and spelling of words, rules of composition, and grammar.” (for level of importance of language arts) for each occupation named by the students, on a scale ranging from 0 (*not at all math-/language-related*) to 100 (*highly math-/language-related*). The interrater reliability was good, κ = 0.82.

#### Self-Reported Achievement

Achievement in mathematics and in the verbal domain was assessed by students’ self-reported school grades at the end of the last semester in the school year. In Germany, school grades range from 1 (*very good*) to 6 (*unsatisfactory*), with lower values indicating better performance. To facilitate the interpretation of the findings, we reverse-coded the grades so that higher values reflect better achievement.

### Statistical Analyses

A longitudinal structural equation modeling approach with a cross-lagged panel design was used, and the same variables were measured across time points (Kenny, 1975). This design enabled us to test the stability of constructs and the bidirectionality of effects between constructs. Three separate models were tested for students’ self-concepts of ability and task values because these constructs tend to be highly correlated: Model 1 included students’ academic self-concept, Model 2 included utility value, and Model 3 included intrinsic value. Each model included the motivational belief variable at Times 1 and 2 (autoregressive path) and career plans at Times 1 and 2 (autoregressive path) in both mathematics and language arts. Students’ gender, immigrant background, school type and self-reported achievement in math and language arts were included as predictors of the Time 2 outcomes in all tested models. Reciprocal associations across Time 1 and Time 2 were tested between the motivational belief variables (academic self-concept, utility value, intrinsic value) and students’ career plans in mathematics and language arts.

Before testing the structural equation models, scalar measurement invariance was tested for the latent variables in the full sample (Byrne, 2004). Scalar measurement invariance (intrinsic value) or partial scalar invariance (self-concept, utility value) was established indicating that the same latent constructs were assessed across time (for more detailed information, see Appendix B). Measurement invariance restrictions were kept when testing the hypothesized effects with longitudinal structural equation modeling. Measurement invariance was also tested across gender (see Appendix B). Mplus 8.0 was used for all analyses (Muthén and Muthén, 1998–2019). The TYPE IS COMPLEX function of Mplus was used to account for the nested structure of the data (students nested within classrooms), and maximum likelihood estimation with robust standard errors (MLR) was applied in all models. Missing data were handled by using full-information maximum likelihood (FIML) estimation. Information about participation rates per school, attrition rate across waves, and missing values on the study variables for each wave are reported in Appendix C. The following criteria were used to evaluate the goodness of fit of the models (Tanaka, 1993): Yuan-Bentler scaled χ^2^ (mean-adjusted test-statistic robust to non-normality), Tucker and Lewis index (TLI), comparative fit index (CFI), and root mean square of approximation (RMSEA) with associated confidence intervals (CIs). Additionally, standardized root mean residual values (SRMR) were reported. TLI and CFI values greater than 0.95, RMSEA values lower than 0.06, and SRMR lower than 0.08 indicate satisfactory model fit (Hu and Bentler, 1999). Indirect effects were tested with the MODEL INDIRECT command and the CINTERVAL option. Bootstrapped standard errors and confidence intervals were obtained to evaluate the estimated indirect effects (Muthén and Muthén, 1998–2019). Indirect effects were estimated based on the product of coefficients method (MacKinnon et al., 2007; Williams and MacKinnon, 2008).

## Results

### Descriptive Statistics and Bivariate Associations

Observed means and standard deviations for all variables included in subsequent analyses are reported in Table 1, and manifest bivariate correlations are reported in Tables 2, 3. These correlational patterns suggest that – both at Time 1 and Time 2 – girls were less likely than boys to report career plans in mathematics (consistent with *Hypothesis 1*) and more likely to report career plans related to the language arts domain (consistent with *Hypothesis 2*). Furthermore, students with comparatively higher self-reported math achievement at Time 1 also reported comparatively higher career plans in mathematics at both Time 1 and Time 2. However, students’ math achievement was also significantly positively related to their career plans in language arts at Time 2. Students’ self-reported achievement in language arts at Time 1 was positively related to their career plans in the math and language arts domains at Time 1 and Time 2. These correlational patterns support positive same-domain associations for achievement and career plans, but no negative cross-domain associations emerged. Achievement is thus positively related to career aspirations across domains.

**Table 1:** Descriptives of the study variables at time 1 (data for time 2 in parentheses) for boys (**n** = 506) and girls (**n** = 594).

	Girls		Boys				

Variable	*M*	*SD*	*M*	*SD*	Wald χ^2^, *df* = 1	*d*	Range
Self-reported mathematics grade	4.03	1.02	3.96	1.23	0.68 n.s.	0.06	1–6
Self-reported grade in language arts	4.45	0.87	4.10	0.85	26.96^∗∗∗^	0.41	1–6
Self-concept in mathematics	3.02 (3.09)	0.87 (0.86)	3.37 (3.45)	0.89 (0.93)	33.70^∗∗∗^(25.26^∗∗∗^)	0.40 (0.40)	1–5
Utility value in mathematics	2.90 (2.92)	0.93 (0.92)	3.16 (3.17)	0.93 (0.96)	11.80^∗∗∗^ (7.48^∗∗^)	0.30 (0.27)	1–5
Intrinsic value in mathematics	2.82 (2.93)	1.09 (1.12)	3.22 (3.19)	1.13 (1.17)	27.30^∗∗∗^ (7.25^∗∗∗^)	0.36 (0.23)	1–5
Self-concept in language arts	3.50 (3.55)	0.79 (0.80)	3.44 (3.38)	0.77 (0.78)	1.68 n.s. (9.08^∗∗^)	0.07 (0.21)	1–5
Utility value in language arts	3.73 (3.74)	0.94 (0.94)	3.60 (3.55)	1.06 (0.99)	2.76 n.s. (4.61^∗^)	0.13 (0.20)	1–5
Intrinsic value in language arts	3.52 (3.55)	1.05 (1.00)	3.25 (3.25)	1.09 (1.04)	12.14^∗∗∗^ (14.43^∗∗∗^)	0.25 (0.29)	1–5
Career plans related to mathematics	42.54 (41.83)	8.91 (9.04)	44.61 (45.41)	9.92 (10.06)	8.38^∗∗^ (17.91^∗∗∗^)	0.22 (0.37)	0–100
Career plans related to language arts	57.65 (57.82)	6.39 (6.19)	55.70 (55.45)	6.67 (6.44)	16.92^∗∗∗^(12.81^∗∗∗^)	0.30 (0.38)	0–100

*Latent means are reported for the latent variables including measurement invariance across time and gender groups. Grades were recoded. ^∗^*p* < 0.05, ^∗∗^*p* < 0.01, ^∗∗∗^*p* < 0.001.*

**Table 2:** Intercorrelations between the study variables.

	1	2	3	4	5	6	7	8	9	10	11	12
1) Girl												
2) German native	–0.04											
3) Lang achiev	0.20***	0.11*										
4) Math achiev	0.03	0.13***	0.46***									
5) Comp. school	–0.13***	–0.06	–0.26***	–0.23***								
6) Int math T1	–0.18***	0.01	0.10**	0.47***	–0.06							
7) Int math T2	–0.12**	0.04	0.12**	0.45***	–0.07	0.71***						
8) Int lang T1	0.12***	–0.01	0.30***	–0.16***	0.01	–0.09*	–0.09*					
9) Int lang T2	0.14***	–0.08*	0.15***	–0.13**	0.01	–0.13***	–0.11***	0.70***				
10) Self-concept math T1	–0.20***	0.01	0.22***	0.65***	–0.11***	0.81***	0.66***	–0.16***	–0.20***			
11) Self-concept math T2	–0.20***	0.06	0.20***	0.56***	–0.09*	0.66***	0.81***	–0.17***	–0.21***	0.80***		
12) Self-concept lang T1	0.04	0.03	0.46 ***	–0.09*	–0.04	–0.13***	–0.15***	0.71***	0.55***	–0.09**	–0.10**	
13) Self-concept lang T2	0.10**	0.04	0.36***	–0.07	–0.05	–0.13***	–0.19***	0.54***	0.71***	–0.10**	–0.11**	0.67***
14) Utility math T1	–0.14**	–0.10**	0.01	0.17***	0.13***	0.52***	0.40***	0.04	0.02	0.44***	0.38***	–0.01
15) Utility math T2	–0.13**	0.07	0.04	0.22***	0.12**	0.40***	0.54***	–0.06	–0.04	0.36***	0.49***	–0.10*
16) Utility lang T1	0.07	–0.08*	0.15***	–0.04	0.10*	0.03	0.04	0.56***	0.38***	–0.03	–0.05	0.40***
17) Utility lang T 2	0.10*	–0.06	0.17***	–0.05	0.09	–0.02	0.02	0.42***	0.54***	–0.09	–0.04	0.34***
18) Career math T1	–0.08*	–0.02	0.09*	0.19***	–0.25***	0.29***	0.24***	–0.08*	–0.10*	0.28***	0.23***	–0.09*
19) Career math T2	–0.12***	–0.03	0.09	0.16**	–0.21***	0.26***	0.23***	–0.10*	–0.07	0.28***	0.27***	–0.08
20) Career lang T1	0.15**	–0.05	0.17***	0.04	–0.25***	–0.04	–0.08	0.11**	0.13***	–0.01	–0.05	0.17***
21) Career lang T2	0.17**	0.01	0.14*	0.08	–0.28***	–0.01	–0.03	0.06	0.07	0.03	–0.02	0.10**

*^∗^p < 0.05, ^∗∗^ p < 0.01,^∗∗∗^p < 0.001; Lang achiev, self-reported grade German; Comp. school, comprehensive school (“Integrierte Sekundarschule”); Math achiev, self-reported grade Mathematics; Int math T1/T1, Intrinsic value in mathematics at Time 1/Time 2; Intl ang T1/T2, Intrinsic value in language arts at Time 1/Time 2; Self-concept maths T1/T2, Self-concept in mathematics at Time 1/Time 2; Self- concept lang T1/T2, Self-concept in language arts at Time 1/Time 2; Utility maths T1/T2, Utility value in mathematics at Time 1/Time 2; Utility lang T1/T2, Utility value in language arts at Time 1/Time 2; Career math T1/T2, Career plans in math-related fields at Time 1/ Time 2; Career lang T1T/2, Career plans in language domain at Time 1/Time 2.*

**Table 3:** Intercorrelations between the study variables – continuation of Table 2.

	13	14	15	16	17	18	19	20	21
14) Utility maths T1	–0.01								
15) Utility maths T2	–0.07	0.59***							
16) Utility lang T1	0.31***	0.18***	0.07						
17) Utility lang T2	0.40***	0.09*	0.09*	0.57***					
18) Career math T1	–0.10*	0.20***	0.12*	–0.11*	–0.10*				
19) Career math T2	–0.13***	0.17***	0.17***	–0.12**	–0.08*	0.63***			
20) Career lang T1	0.15***	–0.11**	–0.12**	0.11***	0.16***	–0.08*	–0.10*		
21) Career lang T2	0.13**	–0.04	–0.10*	0.13***	0.16***	–0.03	–0.06	0.63***	

*^∗^p < 0.05, ^∗∗^p < 0.01, ^∗∗∗^p < 0.001; Utility lang T1/T2, Utility value in language arts at Time 1/Time 2; Career math T1/T2, career plans in math-related fields at Time 1/Time 2; Career lang T1T/2, career plans in language domain at Time 1/Time 2.*

However, positive same-domain and negative cross-domain effects were corroborated for the associations between math-related career plans and math- and language arts-related motivations. Specifically, students’ math-related career plans (at Time 1 and Time 2) were significantly and positively correlated with students’ self-concept, utility value, and intrinsic value in math at both Time 1 and Time 2 (consistent with *Hypothesis 3* in the math domain) and were significantly and negatively correlated with students’ self-concept, utility value, and intrinsic value in language arts at both Time 1 and Time 2 (consistent with *Hypothesis 4* in the math domain). Analogous same-domain associations were confirmed for the language arts domain (consistent with *Hypothesis 3* in the verbal domain). Specifically, language arts-related career plans at Time 1 and Time 2 were significantly and positively correlated with students’ self-concept, utility value and intrinsic value in language arts at Time 1 and Time 2^1^. However, significant negative cross-domain associations were corroborated only for career plans in language arts at Time 1 and Time 2 and utility value in mathematics at Time 1 and Time 2 (only partly consistent with *Hypothesis 4* in the verbal domain). Thus, our expectations were fully supported in the math domain but were only partially supported in the language arts domain. In the following sections, these associations are further examined in the context of cross-lagged structural equations models.

### Students’ Self-Concept, Task Values, and Career Plans in Math and Language Arts

#### Model 1: Self-concept and career plans model

**FIGURE 2:**
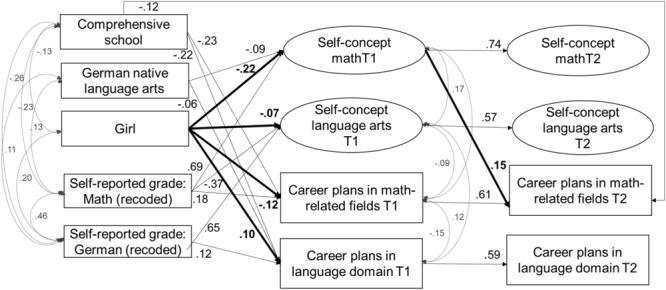
Model 1 – Relations among academic self-concept and career plans in math and language. Standardized and significant (*p* < 0.05) coefficients are depicted.

The model had good fit to the data, χ^2^(211) = 363.13, CFI = 0.98, TLI = 0.98, RMSEA = 0.03, SRMR = 0.02. Standardized and significant coefficients for this model are reported in Figure 2. The standardized coefficients of this model are reported in Tables 4, 5. In line with our expectations (*Hypotheses 1*), girls, relative to boys, reported lower levels of self-concept in mathematics at Time 1 (β = –0.22, *SE* = 0.03, *p* < 0.001) and aspired to occupations that required lower levels of math knowledge at Time 1 (β = –0.12, *SE* = 0.04, *p* = 0.001). Controlling for achievement differences in school grades, girls reported lower levels of self-concept in language arts at Time 1 compared to boys (β = –0.07, *SE* = 0.03, *p* = 0.005), but aspired to occupations that required higher levels of knowledge in language arts than boys (β = 0.10, *SE* = 0.04, *p* = 0.007), in partial support of *Hypothesis 2*. Notably, correlational patters in Tables 2, 3 are fully consistent with *Hypothesis 2*, so that the negative predictive effect of gender on self-concept suggests a larger discrepancy between achievement and self-evaluated abilities for girls than for boys.

**Table 4:** Model 1, Part I: Relations between career plans and academic self-concept.

Variable	Self-concept math T1			Self-concept lang T1			Self-concept math T2			Self-concept lang T2		
	β	*SE*	*p*	β	*SE*	*p*	β	*SE*	*p*	β	*SE*	*p*

Girls	**–0.22**	**0.03**	**<0.001**	**–0.07**	**0.03**	**0.005**	–0.05	0.03	0.076	0.04	0.03	0.121
German native	**–0.09**	**0.03**	**0.001**	0.01	0.03	0.815	0.05	0.03	0.092	0.01	0.03	0.690
Math achiev	**0.69**	**0.03**	**<0.001**	**–0.37**	**0.04**	**<0.001**	0.06	0.04	0.119	–0.02	0.05	0.700
Lang achiev	–0.04	0.04	0.254	**0.65**	**0.04**	**<0.001**	0.03	0.04	0.368	0.09	0.05	0.064
Comp. school	–0.01	0.03	0.964	0.04	0.04	0.360	0.03	0.03	0.310	–0.01	0.03	0.906
Career math T1							0.04	0.03	0.175	–0.03	0.03	0.295
Career lang T1							–0.01	0.02	0.121	0.04	0.02	0.121
Self-concept math T1							**0.74**	**0.03**	**<0.001**	–0.05	0.05	0.379
Self-concept lang T1							–0.04	0.04	0.310	**0.61**	**0.06**	**<0.001**

*N = 1117; German native, German native language; Math achiev, Self-reported grade Mathematics (recoded); Lang achiev, Self-reported grade German (recoded); Comp school, Comprehensive school (“Integrierte Sekundarschule”); Career math T1, career plans in math-related fields at Time 1; Career lang 1, career plans in language domain at Time 1; Self-concept math T1/T2, Self-concept in mathematics at Time 1/Time 2; self-concept lang T1/T2, self-concept in language arts at Time 1/Time 2. Coefficients which are significant at least at *p* < 0.05 are depicted in bold.*

**Table 5:** Model 1, Part II: Relations between academic self-concept and career plans.

	Career math T1			Career lang T1			Career math T2			Career lang T2		
	β	*SE*	*p*	β	*SE*	*p*	β	*SE*	*p*	β	*SE*	*p*

Girls	**–0.12**	**0.04**	**<0.001**	**0.10**	**0.04**	**0.007**	–0.05	0.03	0.121	0.07	0.05	0.138
German native	–0.06	0.04	0.090	**–0.06**	**0.03**	**0.039**	–0.02	0.04	0.682	0.04	0.04	0.365
Math achiev	**0.18**	**0.05**	**<0.001**	–0.05	0.04	0.196	–0.09	0.05	0.090	0.04	0.06	0.578
Lang achiev	–0.02	0.03	0.567	**0.12**	**0.05**	**0.010**	0.06	0.05	0.225	–0.04	0.05	0.444
Comp school	**–0.23**	**0.03**	**<0.001**	**–0.22**	**0.03**	**<0.001**	–0.09	0.05	0.058	**–0.12**	**0.04**	**0.005**
Self math T1							**0.15**	**0.04**	**0.001**	0.03	0.06	0.574
Self lang T1							–0.04	0.04	0.357	0.03	0.04	0.500
Career math T1							**0.57**	**0.05**	**<0.001**	–0.02	0.04	0.634
Career lang T1							–0.06	0.05	0.198	**0.59**	**0.06**	**<0.001**

*N = 1117; German native, German native language; Math achiev, Self-reported grade Mathematics (recoded); Lang achiev, Self-reported grade German (recoded); Comp school, Comprehensive school (“Integrierte Sekundarschule”); Career math T1/T2, career plans in math-related fields at Time 1/ Time 2; Career lang T1/T2, career plans in language domain at Time 1/ Time 2; Self-concept math T1, Self-concept in mathematics at Time 1; self-concept lang T1, self-concept in language arts at Time 1. Coefficients which are significant at least at *p* < 0.05 are depicted in bold.*

Model 1 reveals *positive same-domain effects* but *Hypothesis 3* was supported only in the math domain and not in the language arts domain. These positive same-domain effects were unidirectional from self-concept at Time 1 to career plans at Time 2: Although students’ self-concept in mathematics at Time 1 (ψ = 0.17, *SE* = 0.04, *p* < 0.001) and in language arts at Time 1 (ψ = 0.12, *SE* = 0.04, *p* = 0.001) were significantly and positively correlated with career plans in the respective domain within time, we did not identify significant predictive effects of self-concept in language arts on career plans in language arts across time. Only in mathematics, self-concept at Time1 significantly and positively predicted career plans at Time 2 (β = 0.15, *SE* = 0.04, *p* = 0.001).

Partially confirming our expectations (*Hypothesis 4*), our results also show some *negative cross-domain effects*, but only within time: Students’ self-concept in language arts at Time 1 was significantly and negatively correlated with career plans in math-related fields at Time 1 (ψ = –0.09, *SE* = 0.04, *p* = 0.02). We did not identify significant cross-domain effects between academic self-concept and career plans across time.

Within-time relations suggested positive same-domain effects of achievement on self-concept in mathematics and language arts, and negative cross-domain effects of mathematics achievement on students’ self-concept in language arts, however, we did not find such cross-domain effects across time (*Hypothesis 5*). Students’ beliefs were relatively stable, which may explain the lack of significant longitudinal associations. Although we did not find direct cross-domain effects across time, we were able to identify indirect cross-domain effects of students’ school grade in mathematics at Time 1 on their ability self-concept in language arts at Time 2, mediated via self-concept in language arts at Time 1 (β = –0.20, *SE* = 0.04, *p* < 0.001; 95% CI [–0.27 –0.13]).

In accordance with our expectations (*Hypothesis 6*), there was a significant and indirect effect from student gender to student career plans in math-related fields at Time 2 through student mathematics self-concept – girls reported lower mathematics self-concepts than boys at Time 1, which in turn partially explained their low math-related career plans, β = –0.032, *SE* = 0.01, *p* = 0.02; 95% CI [–0.05 – 0.001].

The following pattern of results emerged for included control variables. Compared to students whose mother tongue was not German, native speakers of German reported lower levels in mathematics self-concept at Time 1 (β = –0.09, *SE* = 0.03, *p* = 0.001). Students in comprehensive schools reported lower career plans in language-related domains at both time points (Time 1: β = -0.12, *SE* = 0.04, *p* = 0.005; Time 2: β = –0.22, *SE* = 0.03, *p* < 0.001) and lower career plans in math-related domains at Time 1 (Time 1: β = -0.23, *SE* = 0.03, *p* < 0.001; Time 2: β = –0.09, *SE* = 0.05, *p* = 0.058) than students in academic track schools. Students’ mathematics achievement at Time 1 positively predicted their mathematics self-concept at Time 1 (β = 0.69, *SE* = 0.03, *p* < 0.001) and their math-related career plans at Time 1 (β = 0.18, *SE* = 0.05, *p* < 0.001). Students’ achievement in language arts at Time 1 positively predicted their self-concept of ability in language arts at Time1 (β = 0.65, *SE* = 0.04, *p* < 0.001) as well as their career plans in the language arts domain at Time 1 (β = 0.12, *SE* = 0.05, *p* = 0.010). The stability of students’ academic self-concept in both mathematics (β = 0.74, *SE* = 0.03, *p* < 0.001) and language arts (β = 0.61, *SE* = 0.06, *p* < 0.001) was relatively high.

The model explained significant amounts of variance in career plans in math-related fields (T1: 10.4%; T2: 42.3%), in language arts-related career plans (T1: 9.1%; T2: 41.3%), as well as in students’ mathematics self-concept (T1: 48.1%; T2: 65.3%) and language arts self-concept (T1: 32.1%; T2: 46.7%).

#### Model 2: Utility value and career plans model

**FIGURE 3:**
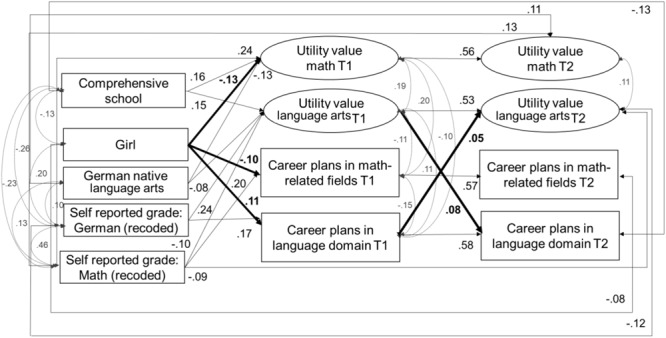
Model 2 – Relations among utility value and career plans in math and language. Standardized and significant (*p* < 0.05) coefficients are depicted.

The model showed a good fit to the data, χ^2^(123) = 152.89, CFI = 0.99, TLI = 0.99, RMSEA = 0.02, SRMR = 0.02. Standardized and significant coefficients for this model are reported in Figure 3. The standardized coefficients of this model are reported in Tables 6, 7. In line with our expectations (*Hypotheses 1*), girls reported comparatively lower levels of utility value in mathematics at Time 1 (β = –0.13, *SE* = 0.03, *p* < 0.001) as well as lower career plans in math-related fields at both time points (Time 1: β = –0.11, *SE* = 0.04, *p* = 0.001; Time 2: β = –0.08, *SE* = 0.04, *p* = 0.038). Also in line with our assumptions (*Hypothesis 2*), girls reported higher career plans in the language domain at Time 1 (β = 0.10, *SE* = 0.04, *p* = 0.011) compared to boys.

**Table 6:** Model 2, Part I: Relations between career plans and utility value.

Variable	Utility math T1			Utility lang T1			Utility math T2			Utility lang T2		
	β	*SE*	*p*	β	*SE*	*p*	β	*SE*	*p*	β	*SE*	*P*

Girls	**–0.13**	**0.03**	**<0.001**	0.06	0.04	0.168	–0.03	0.04	0.441	0.03	0.03	0.437
German native	**–0.13**	**0.04**	**<0.001**	**–0.08**	**0.04**	**0.041**	**0.11**	**0.03**	**0.001**	–0.02	0.04	0.507
Math achiev	**0.24**	**0.04**	**<0.001**	**–0.11**	**0.04**	**0.007**	**0.13**	**0.04**	**0.002**	**–0.09**	**0.03**	**0.004**
Lang achiev	–0.02	0.04	0.629	**0.24**	**0.04**	**<0.001**	–0.01	0.04	0.838	**0.12**	**0.04**	**0.005**
Compr school	**0.16**	**0.05**	**0.001**	**0.15**	**0.04**	**0.001**	0.07	0.04	0.066	0.03	0.03	0.317
Career math T1							–0.01	0.04	0.704	–0.05	0.03	0.130
Career lang T1							–0.04	0.03	0.114	**0.05**	**0.03**	**0.037**
Utility math T1							**0.56**	**0.04**	**<0.001**	0.02	0.04	0.605
Utility lang T1							–0.02	0.04	0.598	**0.53**	**0.04**	**<0.001**

*N = 1117; German native, German native language; Math achiev, Self-reported grade Mathematics (recoded); Lang achiev, Self-reported grade German (recoded); Comp school, Comprehensive school (“Integrierte Sekundarschule”); Career math T1, career plans in math-related fields at Time 1; Career lang T1, career plans in language domain at Time 1; Utility math T1/T2, Utility value in mathematics at Time 1/Time 2 Utility lang T1/T2, Utility value in language arts at Time 1/Time 2. Coefficients which are significant at least at *p* < 0.05 are depicted in bold.*

**Table 7:** Model 2, Part II: Relations between utility value and career plans.

	Career math T1			Career lang T1			Career math T2			Career lang T2		
	β	*SE*	*p*	β	*SE*	*p*	β	*SE*	*P*	β	*SE*	*P*

Girls	**–0.11**	**0.04**	**0.001**	**0.10**	**0.04**	**0.011**	**–0.08**	**0.04**	**0.038**	0.06	0.05	0.173
German native	–0.06	0.04	0.083	**–0.06**	**0.03**	**0.033**	0.05	0.04	0.220	–0.03	0.04	0.446
Math achiev	**0.18**	**0.05**	**<0.001**	–0.05	0.04	0.176	0.01	0.05	0.909	0.05	0.04	0.216
Lang achiev	–0.02	0.03	0.491	**0.13**	**0.05**	**0.007**	0.04	0.05	0.429	–0.05	0.05	0.264
Comp school	**–0.23**	**0.03**	**<0.001**	**–0.22**	**0.03**	**<0.001**	–0.08	0.05	0.232	**–0.13**	**0.04**	**0.001**
Utility math T1							0.06	0.04	0.163	0.03	0.05	0.486
Utility lang T1							–0.06	0.03	0.054	**0.08**	**0.04**	**0.047**
Career math T1							**0.57**	**0.05**	**<0.001**	–0.02	0.04	0.729
Career lang T1							–0.05	0.05	0.291	**0.58**	**0.04**	**<0.001**

*N = 1117; Career math T1/T2, career plans in math-related fields at Time 1/ Time 2; Career lang T1/T2, career plans in language domain at Time 1/ Time 2; German native, German native language; Math achiev, Self-reported grade Mathematics (recoded); Lang achiev, Self-reported grade German (recoded); Comp school, Comprehensive school (“Integrierte Sekundarschule”); Utility math T1, Utility value in mathematics at Time 1; Utility lang T1 = Utility value in language arts at Time 1. Coefficients which are significant at least at *p* < 0.05 are depicted in bold.*

Consistent with *Hypothesis 3*, we identified *positive same domain effects*, but only for language arts and not for mathematics: We identified positive same-domain effects between utility value and career plans across time for language arts; utility value in language arts at Time 1 positively predicted career plans in language-related domains at Time 2 (β = 0.08, *SE* = 0.04, *p* = 0.047). Our assumptions about *cross-domain effects* between motivational beliefs and career plans (*Hypothesis 4*) were not confirmed for utility value longitudinally: neither utility value in language arts at Time 1 predicted career plans in mathematics at Time 2 (β = –0.02, *SE* = 0.03, *p* = 0.054) nor did utility value in mathematics at Time 1 predict career plans in language-related domains at Time 2 (β = 0.03, *SE* = 0.05, *p* = 0.486).

In line with our expectations (*Hypothesis* 5), we found positive same-domain effects of achievement at Time 1 on utility value at Time 2 for both mathematics and language arts, and a negative cross-domain effect of mathematics achievement at Time 1 on students’ utility value in language arts at Time 2. Although we did not find direct negative cross-domain effects for the relation between self-reported achievement and utility value across time, we were able to identify indirect cross-domain effects of students’ self-reported grade in mathematics at Time 1 on utility value in language arts at Time 2 via utility value in language arts at Time 1 (β = –0.05, SE = 0.02, *p* = 0.007; 95% CI [–0.08 –0.01]).

Contrary to expectations about gender-specific (same-domain and cross-domain) motivational processes (*Hypothesis 6*), we did not find any significant indirect effects from gender on career plans via utility value (language arts: β = 0.01, *SE* = 0.01, *p* = 0.28; 95% CI [–0.05 0.17]; mathematics: β = -0.01, *SE* = 0.01, *p* = 0.18; 95% CI [–0.38 0.07]).

With regard to our control variables, we found that, compared to students whose mother tongue was not German, students whose mother tongue was German reported lower utility value in mathematics (β = –0.13, *SE* = 0.04, *p* < 0.000) and language arts (β = –0.08, *SE* = 0.04, *p* = 0.041) at Time 1, but higher utility value in mathematics at Time 2 (β = 0.11, *SE* = 0.03, *p* < 0.001). Students in comprehensive schools reported higher utility value of language-related domains (β = 0.15, *SE* = 0.04, *p* < 0.001) and math (β = 0.16, *SE* = 0.05, *p* < 0.001) at Time 1. Students’ mathematics achievement at Time 1 positively predicted their utility value in mathematics at Time 1 (β = 0.24, *SE* = 0.04, *p* < 0.001) and Time 2 (β = 0.13, *SE* = 0.04, *p* = 0.002), as well as their math-related career plans at Time 1 (β = 0.18, *SE* = 0.05, *p* < 0.001). Students’ achievement in language arts at Time 1 positively predicted their utility value in language arts at Time 1 (β = 0.24, *SE* = 0.04, *p* < 0.001) and Time 2 (β = 0.12, *SE* = 0.04, *p* = 0.005) as well as their career plans in the language arts domain at Time 1 (β = 0.13, *SE* = 0.05, *p* = 0.007). The stability of reported utility value in both mathematics (β = 0.56, *SE* = 0.04, *p* < 0.001) and language arts (β = 0.53, *SE* = 0.04, *p* < 0.001) was relatively high across both time points.

The model explained significant amounts of variance in career plans in math-related fields (T1: 10.3%; T2: 40.7%), career plans in language arts-related fields (T1: 9.1%; T2: 41.9%), as well as in mathematics utility value (T1: 9.7%; T2: 38.3%) and language arts utility value (T1: 6.7%; T2: 35.0%).

#### Model 3: Intrinsic value and career plans model

**FIGURE 4:**
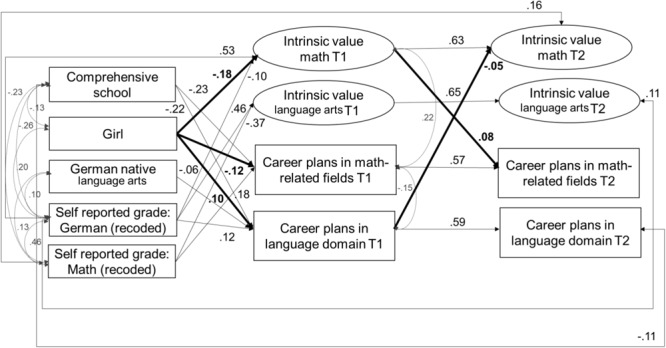
Model 3 – Relations among intrinsic value and career plans in math and language. Standardized and significant (*p* < 0.05) coefficients are depicted.

The model showed a good fit to the data, χ^2^(124) = 192.19, CFI = 0.99, TLI = 0.98, RMSEA = 0.02, SRMR = 0.02. Standardized and significant coefficients for this model are reported in Figure 4. The standardized coefficients of this model are reported in Tables 8, 9. In line with our assumptions (*Hypothesis 1*), girls relative to boys reported lower levels of intrinsic value in mathematics at Time 1 (β = –0.18, *SE* = 0.03, *p* < 0.001) and were less likely to report career plans in math-related fields at Time 1 (β = –0.11, *SE* = 0.04, *p* = 0.001). Also in line with our expectations (*Hypothesis 2*), girls were more likely than boys to report career plans in language arts domains at Time 1 (β = 0.10, *SE* = 0.04, *p* = 0.009). The stability of students’ intrinsic value in both mathematics (β = 0.63, *SE* = 0.04, *p* < 0.001) and language arts (β = 0.65, *SE* = 0.04, *p* < 0.001) was high across the two time points.

**Table 8:** Model 3, Part I: Relations between career plans and intrinsic value.

Variable	Intrinsic math T1			Intrinsic lang T1			Intrinsic math T2			Intrinsic lang T2		

	β	*SE*	*p*	β	*SE*	*p*	β	*SE*	*p*	β	*SE*	*P*
Girls	**–0.18**	**0.03**	**<0.001**	0.05	0.03	0.125	0.01	0.03	0.871	0.02	0.03	0.491
German native	–0.06	0.03	0.093	–0.01	0.03	0.945	0.02	0.03	0.405	0.02	0.04	0.549
Math achiev	**0.53**	**0.04**	**<0.001**	**–0.36**	**0.02**	**<0.001**	**0.16**	**0.05**	**0.002**	–0.07	0.04	0.091
Lang achiev	**–0.10**	**0.04**	**0.017**	**0.47**	**0.03**	**<0.001**	–0.01	0.05	0.862	**0.11**	**0.05**	**0.025**
Compr school	0.01	0.04	0.971	0.06	0.05	0.228	0.01	0.03	0.769	0.01	0.03	0.709
Career math T1							0.03	0.03	0.414	–0.03	0.03	0.391
Career lang T1							**–0.05**	**0.02**	**0.047**	0.03	0.03	0.213
Intrinsic math T1							**0.63**	**0.04**	**<0.001**	–0.04	0.04	0.298
Intrinsic lang T1							–0.01	0.04	0.917	**0.65**	**0.03**	**<0.001**

*N = 1117; German native, German native language; Math achiev, Self-reported grade Mathematics (recoded); Lang achiev, Self-reported grade German (recoded); Comp school, Comprehensive school (“Integrierte Sekundarschule”); Career math T1, career plans in math-related fields at Time 1; Career lang 1, career plans in language domain at Time 1; Intrinsic math T1/ T2, Intrinsic value in mathematics at Time 1/Time 2; Intrinsic lang T1/T2, Intrinsic value in language arts at Time 1/Time 2. Coefficients which are significant at least at *p* < 0.05 are depicted in bold.*

**Table 9:** Model 3, Part II: Relations between intrinsic value and career plans.

	Career math T1			Career lang T1			Career math T2			Career lang T2		

	β	*SE*	*p*	β	*SE*	*p*	β	*SE*	*p*	β	*SE*	*p*
Girls	**–0.11**	**0.04**	**0.001**	**0.10**	**0.04**	**0.011**	–0.06	0.04	0.076	0.06	0.05	0.185
German native	–0.06	0.04	0.083	**–0.06**	**0.03**	**0.033**	0.03	0.04	0.477	0.04	0.04	0.344
Math achiev	**0.18**	**0.05**	**<0.001**	–0.05	0.04	0.176	–0.03	0.05	0.470	0.05	0.05	0.351
Lang achiev	–0.02	0.03	0.491	**0.13**	**0.05**	**0.007**	0.06	0.05	0.222	–0.03	0.05	0.546
Comp school	**–0.23**	**0.03**	**<0.001**	**–0.22**	**0.03**	**<0.001**	–0.09	0.05	0.058	**–0.11**	**0.04**	**0.007**
Intrinsic math T1							**0.08**	**0.03**	**0.020**	0.03	0.05	0.486
Intrinsic lang T1							–0.06	0.04	0.173	0.01	0.05	0.844
Career math T1							**0.57**	**0.05**	**<0.001**	–0.02	0.04	0.640
Career lang T1							–0.05	0.05	0.263	**0.59**	**0.05**	**<0.001**

*N = 1117; Career math T1/T2, career plans in math-related fields at Time 1/ Time 2; Career lang T1/T2, career plans in language domain at Time 1/Time 2; German native, German native language; Math achiev, Self-reported grade Mathematics (recoded); Lang achiev, Self-reported grade German (recoded); Comp school, Comprehensive school (“Integrierte Sekundarschule”); Intrinsic math T1, Intrinsic value in mathematics at Time 1; Intrinsic lang T1, Intrinsic value in language arts at Time 1. Coefficients which are significant at least at *p* < 0.05 are depicted in bold.*

Our assumptions about *positive same-domain effects*: (*Hypothesis 3*) were –, similarly, to our results for self-concept – only confirmed for the domain of mathematics, but not for language arts: Intrinsic value in mathematics at Time 1 significantly and positively predicted career plans in math-related fields at Time 2 (β = 0.08, *SE* = 0.03, *p* = 0.020). The effect was unidirectional as intrinsic value positively predicted career plans (and not vice versa).

In accordance with our expectations (*Hypothesis 4*), we also identified *cross-domain effects*: Intrinsic value in mathematics at Time 2 significantly and negatively predicted by career plans in language arts at Time 1 (β = –0.05, *SE* = 0.02, *p* = 0.047). This effect was unidirectional: interestingly, career plans predicted subsequent intrinsic value (and not vice versa).

Partially confirming our expectations (*Hypothesis 5*), we found positive same-domain effects of mathematics achievement at Time 1 on mathematics intrinsic value at Time 2, and of achievement in language-arts at Time 1 on intrinsic value in language-arts at Time 2. Although we did not find direct negative cross-domain effects for the relation between self-reported achievement and intrinsic value across time, we were able to identify indirect cross-domain effects from self-reported grade in mathematics at Time 1 on intrinsic value in language arts at Time 2 via intrinsic value in language arts at Time 1 (β = –0.22, *SE* = 0.02, *p* < 0.001; 95% CI [– 0.27 – 0.17]) and from self-reported grade in German at Time 1 on mathematics intrinsic value at Time 2 via mathematics intrinsic value at Time 1 (β = -0.05, *SE* = 0.02, *p* = 0.03; 95% CI [–0.09 –0.01]).

Gender-specific motivational processes (see *Hypothesis 6*) were identified only for mathematics: There was a significant and indirect effect from student gender to student career plans in math-related fields at Time 2, which was mediated via students’ intrinsic valuing of mathematics – girls reported lower mathematics intrinsic value than boys at Time 1, which in turn corresponded to a lower probability of pursuing math-related careers, β = –0.014, *SE* = 0.01, *p* = 0.03; 95% CI [– 0.03 – 0.01]. This effect size, however, was very small.

With regard to our control variables, we found that compared to students whose mother tongue was not German, students whose mother tongue was German had a lower likelihood of striving for careers related to language arts (β = –0.06, *SE* = 0.03, *p* = 0.033) at Time 1. Students in comprehensive schools were less likely to strive for careers in math at Time 1 (Time 1: β = –0.23, *SE* = 0.03, *p* < 0.001) or for careers related to language arts at Time 1 and Time 2 (Time 1: β = –0.22, *SE* = 0.03, *p* < 0.001; Time 2: β = –0.11, *SE* = 0.04, *p* = 0.007). Students’ mathematics achievements at Time 1 positively predicted their intrinsic value in mathematics at Time 1 (β = 0.53, *SE* = 0.04, *p* < 0.001) and Time 2 (β = 0.16, *SE* = 0.05, *p* = 0.002), as well as math-related career plans at Time 1 (β = 0.18, *SE* = 0.05, *p* < 0.001). Students’ achievement in language arts at Time 1 positively predicted their intrinsic value in language arts at Time 1 (β = 0.47, *SE* = 0.03, *p* < 0.001) and Time 2 (β = 0.11, *SE* = 0.05, *p* = 0.003), as well as their language arts-related career plans at Time 1 (β = 0.12, *SE* = 0.05, *p* = 0.007). Students’ mathematics achievements at Time 1 negatively predicted their intrinsic valuing of language arts at Time1 (β = –0.36, *SE* = 0.03, *p* < 0.001). Students’ achievement in language arts at Time 1 negatively predicted their intrinsic valuing of mathematics at Time 1 (β = – 0.10, *SE* = 0.04, *p* = 0.012). Career plans in mathematics at Time 1 were significantly and positively correlated with intrinsic value in mathematics at Time 1 (ψ = 0.22, *SE* = 0.04, *p* < 0.001) and were significantly and negatively correlated with career plans in language arts at Time 1 (ψ = –0.15, *SE* = 0.03, *p* < 0.001). The model explained significant amounts of variance in career plans in math-related fields (T1: 10.2%; T2: 41.5%), in language arts-related fields (T1: 9.1%; T2: 41.1%), as well as in intrinsic value in math (T1: 27.3%; T2: 53.5%) and intrinsic value in language arts (T1: 20.5%; T2: 51.1%).

In a set of supplemental analyses reported in Appendix D, we included math- and language arts-related academic self-concept, intrinsic and utility values, and career plans in one model. The results of this model show negative cross-domain effects of self-concept on task values. Self-concept in mathematics at Time 1 negatively predicts intrinsic value in language-arts at Time 2 (β = -0.18, *SE* = 0.07, *p* = 0.010). Self-concept in language-arts at Time 1 negatively predicts intrinsic value in mathematics (β = -0.13, *SE* = 0.04, *p* = 0.003) and utility value in mathematics (β = -0.10, *SE* = 0.04, *p* = 0.019) both at Time 2. Thus, our findings confirm the key role of academic self-concept in dimensional comparison effects and show that these effects apply to students’ task values as well (see Gaspard et al., 2018).

Our additional results also show positive same-domain effects of self-concept and intrinsic value. Self-concept in mathematics at Time 1 positively predicts intrinsic value in mathematics at Time 2. Intrinsic value in language-arts at Time 1 positively predicts self-concept in language-arts (β = 0.16, *SE* = 0.06, *p* = 0.010) and utility value in language-arts (β = 0.10, *SE* = 0.05, *p* = 0.036) both at Time 2. Thus, the same-domain effects that we show confirm reciprocal links between academic self-concept and intrinsic value within-domains.

## Discussion

This study investigated whether dimensional comparison processes regarding girls’ and boys’ motivational beliefs might contribute to gendered career plans in mathematics and language arts. Furthermore, we investigated whether motivational beliefs and career plans were reciprocally related across two academic domains. Our findings revealed systematic gender differences in *same domain effects* in mathematics: girls’ comparatively lower mathematics self-concept and intrinsic value predicted a lower likelihood of striving for a math-related career. Furthermore, and contrary to expectations, *cross-domain effects* were not related to gender-specific career plans, with only one exception. Girls’ lower levels of intrinsic value in mathematics corresponded to a higher likelihood of striving for a career in language-related fields, which subsequently predicted lower levels of intrinsic value in mathematics. This finding points to a need to address both gender-specific motivational beliefs and gender-specific career plans in school when aiming to enhance more gender equality in girls’ and boys’ occupational choices.

### Gendered Motivational Beliefs and Career Plans in Math and Language-Related Domains

Our hypotheses regarding gender differences in motivational beliefs and career plans of students in math and language-related domains were mostly confirmed. Consistent with prior evidence (Marsh and Yeung, 1998; Watt, 2004; Watt et al., 2012; Gaspard et al., 2015), girls reported lower academic self-concept, intrinsic and utility values in mathematics than boys, and were less likely than boys to strive for careers in math-intensive fields (*Hypothesis 1*). This is notable, given that girls and boys did not differ substantially in terms of self-reported mathematics achievement. Thus, despite gender equality in grades in mathematics, girls felt less competent than did boys in math. Notably, grades are quite important in this context, because they are one of the main factors determining access to higher education, including in the fields of math and science. Yet, negative self-beliefs reduce the likelihood of pursuing math-intensive careers, even when access is possible. Stereotype threat effects might contribute to this discrepancy between achievement feedback and self-perceptions (Steele, 1997). If teachers or parents communicate, for example, through their achievement-related expectations and feedback behaviors that mathematics is a subject that is “typically male” (Tiedemann, 2002; Tenenbaum and Leaper, 2003), girls can feel less competent in the subject despite their high achievement. Our findings point to the need to foster girls’ self-concept in mathematics, for example, by providing them with positive feedback about their intellectual performance in math classes (Dweck, 1978).

Our hypotheses about gender differences in language-related fields were only partly confirmed. Without taking into account gender differences in self-reported grades, boys reported lower self-concept and lower utility value than girls in language arts at the middle of the school year, but not at the beginning of the school year. Furthermore, boys reported comparatively lower levels of intrinsic value in language arts. However, when differences in achievement were controlled (girls had higher grades in language arts than boys), girls reported lower self-concept in language arts than boys already at the beginning of the school year. Controlling for grades, there was no longer a statistically significant effect of gender on utility value or intrinsic value in language arts. This finding extends previous research, which has shown that boys report lower levels of interest and competence beliefs in language arts (Jacobs et al., 2002; Watt, 2004). Gender differences in the language arts domain in the present study appeared to be explained to a large degree by differences in teacher-graded achievement, with the exception of differences in students’ self-concept. Analyses of motivational differences between girls and boys need to take into account achievement differences as well.

### Same-Domain and Cross-Domain Effects

In line with our expectations (*Hypothesis 3*) and based on EVT (Eccles et al., 1983), we found positive same-domain associations between motivational beliefs and career plans. Our expectations were only partially confirmed as we found positive same-domain effects mainly for mathematics. Unidirectional effects were identified showing that academic self-concept and intrinsic value in mathematics predicted subsequent career plans in mathematics-related fields but not vice versa. This result deviates from the reciprocal effects reported by Lauermann et al. (2017). However, whereas Lauermann et al. (2017) asked students about their subjective probability of pursuing careers in math and science, in our study, the importance of math was inferred from open-ended reports of desired careers. If students are not fully aware of the degree to which mathematics is important for their career choice, the predictive power of such choices for subsequent motivations may be reduced. Accordingly, the degree to which students are aware of academic requirements that are relevant for attaining particular careers may play an important moderating role in these reciprocal links.

Most studies that have examined same-domain effects among motivational beliefs and career plans have focused on the domain of mathematics (Lauermann et al., 2017; Lazarides et al., 2017; Wang, 2012; Watt et al., 2012) or science (Guo et al., 2017). Only few studies have examined same-domain effects among motivational beliefs and career plans in domains that are stereotyped as typically female (Lauermann et al., 2015). Building on such previous findings, our study showed that when focusing on the single components of the task value construct, we only found such positive same domain effects for utility value in the domain of language arts. Thus, we find differential effects among the task value components depending on the domain at hand. In mathematics, intrinsic value, and academic self-concept were important predictors of subsequent career plans, whereas in language arts, utility value emerged as a significant predictor of subsequent language-related career plans. Mathematics is often stereotyped as being difficult and not interesting (for mathematics: Watson et al., 1994), which may explain why students’ ability beliefs and interest emerged as significant predictors in this domain. It may be that only students who are highly interested and who feel highly competent in math might tend to strive for careers in math-intensive fields. By comparison, in language-related fields, in which students in our sample were more likely to feel competent and interested, the utility of the domain was more important for their career plans.

In line with the dimensional comparison theory (Möller and Köller, 2001), we identified a set of negative cross-domain effects (*Hypothesis 4*). Specifically, we found one negative cross-domain effect for language-related career plans that negatively predicted subsequent mathematics intrinsic value. If students strived for a career in language-related domains (e.g., writer, journalist) at the beginning of the school year, they reported lower intrinsic value for math at the middle of the school year. Thus, students’ career plans in language-related domains seemed to initiate specialization processes and led to a reduction of interest in domains that would not help students to achieve their goals.

Interestingly, we identified the expected positive same-domain effects across time for the relation between achievement and task values in mathematics and language arts, but not for the relation between achievement and academic self-concept. We also identified a direct negative cross-domain effect of mathematics achievement (Time 1) on utility value in language arts (Time 2), but not for academic self-concept. However, academic self-concept in our study was highly stable from the beginning of the school year to mid-year. Focusing on a longer time span might be necessary to adequately capture changes in students’ beliefs over time, as proposed in the I/E model (Marsh, 1986).

### Gendered Career Plans and Dimensional Comparison Processes

One central contribution of our study to previous work is that we examined whether and how both same- and cross-domain effects contributed to gendered career plans in mathematics- and language-related fields. Few studies have examined the potential interrelations between student gender and dimensional comparison effects in predicting students’ educational (Nagy et al., 2006; Parker et al., 2012; Guo et al., 2017) and career plans and choices (Wang et al., 2013; Lauermann et al., 2015). Their findings showed that dimensional comparison processes can explain gendered educational and career plans in certain domains. Focusing on math vs. language-related career plans, our study only partially confirmed our expectations about gender-specific (same-domain and cross-domain) motivational processes (*Hypothesis 6*). Only for same-domain effects we found a significant indirect effect of gender on career plans through student motivational beliefs. Girls reported lower mathematics self-concept and intrinsic value at time 1 than boys and were subsequently less likely to strive for careers in math-related fields at time 2. We did not find such effects for language-related motivational beliefs mainly because gender differences in motivational beliefs in language-related domains were not as pronounced as they were in mathematics. This might also be an explanation for the different findings in our study compared to previous studies that found such negative cross-domain effects, for example, for gendered task value and career plans in the field of human-services occupations (Lauermann et al., 2015). Another explanation might be the longitudinal design of the current study. Negative cross-domain effects might have emerged if we had not controlled for prior beliefs because both beliefs and career plans were relatively stable. However, the longitudinal design is an important strength of the present study, as it allows us to examine the effects of motivational beliefs on potential changes in students’ career plans within the school year (and vice versa), and thus, applies a developmental perspective. A longer period of time, however, may need to be considered to examine how and why these beliefs may influence each other over time.

Our findings of differential effects for math and language-related domains point to a need to consider the interrelations between learning contexts and student characteristics. Thus, especially in mathematics, girls’ lower self-concept and intrinsic value seem to be an explanation for gendered career plans in corresponding fields, whereas boys’ lower intrinsic value in language arts did not explain their lower likelihood of striving for careers in language-related domains. More studies are needed that investigate the factors that contribute to boys’ lower likelihood of striving for careers in fields that are stereotyped as “typically female.” Such factors could be related to the matching of the image of these domains and boys’ self-identity (Kessels et al., 2006).

### Limitations

Our study has several limitations that need to be discussed when interpreting its findings. First, the operationalization of career plans is referring to concrete ideas more than to aspirations as students were asked “What job do you think are you going to have when you are 30 years old?” This needs to be considered when comparing the results of this study to previous studies that asked students, for example, for the likelihood of pursuing a career in a certain field (Lauermann et al., 2017). Furthermore, it is an important question whether language-related careers are specific enough as an outcome variable as many careers require verbal and communication-related skills. There are careers that require substantially higher language skills and knowledge than mathematical skills and knowledge (e.g., media consultant and journalist), so that the category “language-relatedness” can be meaningful for a specific group of occupations. However, other careers that are typically considered to be math-intensive (e.g., astrophysicist or mathematician) require very high levels of math and verbal skills and knowledge. Our data preclude us from examining potential discrepancies between required levels of domains-specific skills and knowledge across occupations and students’ subjective beliefs about these occupations. It is also important to note that we were unable to examine potential gaps between students’ career aspirations and educational goals. Schneider and Stevenson (1999) point out, for example, that adolescents with clear understandings of the amount of education needed for their aspired careers are more likely to achieve their aspirations. Thus, future studies need to assess not only adolescents’ occupational aspirations but also corresponding educational goals. Lastly, cross-lagged panel studies have recently been criticized because of their inability to differentiate between relatively stable between-person differences and within-person developmental processes (Hamaker et al., 2015). A larger number of time points, and potentially a larger longitudinal sample than the one available for this study would be necessary for systematic analyses of longitudinal state- and trait-level differences.

## Conclusion

Taken together, our findings corroborate previous research (Watt et al., 2012; Lazarides and Watt, 2015; Lauermann et al., 2017) by showing that gender differences in academic motivations contribute to gendered career plans in mathematics (*same-domain effects*), but, we did not find analogous effects for language arts. In addition, negative *cross-domain effects* did not significantly explain gendered career plans in language-related domains. However, language-related career plans negatively predicted students’ intrinsic valuing of mathematics, which in turn predicted a decrease in language-related career plans. For educational practice, our findings suggest that it is likely important for teachers to enhance interest and self-concept of girls in mathematics, but also to directly speak with boys and girls about their career plans in specific fields.

## Ethics Statement

This study was carried out in accordance with the recommendations of the Berlin Senate for Education, Science and Research (senbwf) with written informed consent from all subjects. All subjects gave written informed consent in accordance with the Declaration of Helsinki. The protocol was approved by the responsible committee at senbwf.

## Author Contributions

RL conducted the analyses and collected the data, and drafted the manuscript. FL contributed decisively to the development of the manuscript.

## Conflict of Interest Statement

The authors declare that the research was conducted in the absence of any commercial or financial relationships that could be construed as a potential conflict of interest.

## Footnotes

1 The correlation between Time 1 intrinsic value and Time 2 career plans was positive but failed to reach significance.

## References

[B1] Arens A. K. Jansen M. (2016). Self-concepts in reading, writing, listening, and speaking: a multidimensional and hierarchical structure and its generalizability across native and foreign languages. *J. Educ. Psychol.* 108 646–664. 10.1037/edu0000081

[B2] Brunner M. Lüdtke O. Trautwein U. (2008). The internal/external frame of reference model revisited: incorporating general cognitive ability and general academic self-concept. *Multivar. Behav. Res.* 43 137–172. 10.1080/00273170701836737 26788975

[B3] Byrne B. M. (2004). Testing for multigroup invariance using AMOS graphics: a road less traveled. *Struct. Equa. Model.* 11 272–300. 10.1207/s15328007sem1102_8

[B4] Dickhäuser O. Reuter M. Hilling C. (2005). Coursework selection: a frame of reference approach using structural equation modelling. *Br. J. Educ. Psychol.* 75 673–688. 10.1348/000709905x37181 16318685

[B5] Durik A. M. Vida M. Eccles J. S. (2006). Task values and ability beliefs as predictors of high school literacy choices: a developmental analysis. *J. Educ. Psychol.* 98 382–393. 10.1037/0022-0663.98.2.382

[B6] Dweck C. S. (1978). Sex differences in learned helplessness: II. the contingencies of evaluative feedback in the classroom and III. An experimental analysis. *Dev. Psychol.* 14 258–278. 10.1037//0012-1649.14.3.268

[B7] Eccles J. S. (2005). “Subjective task value and the Eccles et al. model of achievement-related choices,” in *Handbook of Competence and Motivation* eds Elliot A. J. Dweck C. S. (New York, NY: Guilford) 105–131.

[B8] Eccles J. S. (2009). Who am I and what am I going to do with my life? personal and collective identities as motivators of action. *Educ. Psychol.* 44 78–89. 10.1080/00461520902832368

[B9] Eccles J. S. Adler T. F. Futterman R. Goff S. B. Kaczala C. M. Meece J. (1983). “Expectancies, values and academic behaviors,” in *Achievement and Achievement Motives: Psychological and Sociological Approaches* ed. Spence J. T. (San Francisco, CA: Freeman) 75–146.

[B10] Eccles J. S. Wigfield A. (1995). In the mind of the actor: The structure of adolescents’ achievement task values and expectancy-related beliefs. *Pers. Soc. Psychol. Bull.* 21 215–225. 10.1177/0146167295213003

[B11] Eccles J. S. Wigfield A. (2002). Motivational beliefs, values, and goals. *Annu. Rev. Psychol.* 53 109–132. 10.1146/annurev.psych.53.100901.135153 11752481

[B12] Eccles J. S. Wigfield A. Schiefele U. (1998). “Motivation to succeed,” in *Handbook of Child Psychology* 5 Edn Vol. 3 ed. Eisenberg N. (New York, NY: Wiley) 1017–1095.

[B13] Frenzel A. C. Goetz T. Pekrun R. Watt H. M. G. (2010). Development of mathematics interest in adolescence: Influences of gender, family, and school context. *J. Res. Adolesc.* 20 507–537. 10.1111/j.1532-7795.2010.00645.x

[B14] Gaspard H. Dicke A.-L. Flunger B. Schreier B. Häfner I. Trautwein U. (2015). More value through greater differentiation: gender differences in value beliefs about math. *J. Educ. Psychol.* 107 663–677. 10.1037/edu0000003

[B15] Gaspard H. Wigfield A. Jiang Y. Nagengast B. Trautwein U. Marsh H. W. (2018). Dimensional comparisons: how academic track students’ achievements are related to their expectancy and value beliefs across multiple domains. *Contemp. Educ. Psychol.* 52 1–14. 10.1016/j.cedpsych.2017.10.003

[B16] Guo J. Marsh H. W. Parker P. D. Morin A. J. Dicke T. (2017). Extending expectancy-value theory predictions of achievement and aspirations in science: dimensional comparison processes and expectancy-by-value interactions. *Learn. Instruct.* 49 81–91. 10.1016/j.learninstruc.2016.12.007

[B17] Hamaker E. L. Kuiper R. M. Grasman R. P. (2015). A critique of the cross-lagged panel model. *Psychol. Methods* 20 102–116. 10.1037/a0038889 25822208

[B18] Hu L. Bentler P. M. (1999). Cutoff criteria for fit indexes in covariance structure analysis: conventional criteria versus new alternatives. *Struct. Equ. Model. Multidiscip. J.* 6 1–55. 10.1080/10705519909540118

[B19] Jacobs J. E. Lanza S. Osgood D. W. Eccles J. S. Wigfield A. (2002). Changes in children’s self-competence and values: gender and domain differences across grades one through twelve. *Child Dev.* 73 509–527. 10.1111/1467-8624.00421 11949906

[B20] Kenny D. A. (1975). Cross-lagged panel correlation: a test for spuriousness. *Psychol. Bull.* 82 887–903. 10.1037/0033-2909.82.6.887 7108742

[B21] Kessels U. Rau M. Hannover B. (2006). What goes well with physics? measuring and altering the image of science. *Br. J. Educ. Psychol.* 76 761–780. 10.1348/000709905x59961 17094885

[B22] Lauermann F. Chow A. Eccles J. S. (2015). Differential effects of adolescents’ expectancy and value beliefs about math and english on math/science-related and human services-related career plans. *Int. J. Gender Sci. Technol.* 7 205–228. 10.1111/jora.12218 28453201 PMC5856231

[B23] Lauermann F. Tsai Y.-M. Eccles J. (2017). Math-related career aspirations and choices within eccles et al.’s expectancy–value theory of achievement-related behaviors. *Dev. Psychol.* 53 1540–1559. 10.1037/dev0000367 28639806

[B24] Lazarides R. Dicke A.-L. Rubach C. Eccles J. S. (2019). Profiles of motivational beliefs in math: exploring their development, relations to student-perceived classroom characteristics and impact on future career aspirations and choices. *J. Educ. Psychol.* 28639806

[B25] Lazarides R. Rubach C. Ittel A. (2017). Adolescents’ perceptions of socializers’ beliefs, career-related conversations, and motivation in mathematics. *Dev. Psychol.* 53 525–539. 10.1037/dev0000270 28230404

[B26] Lazarides R. Watt H. M. G. (2015). Student-perceived mathematics teacher beliefs, math classroom learning environments and gendered math career intentions. *Contemp. Educ. Psychol.* 41 51–61. 10.1016/j.cedpsych.2014.11.005

[B27] MacKinnon D. P. Fritz M. S. Williams J. Lockwood C. M. (2007). Distribution of the product confidence limits for the indirect effect: program PRODCLIN. *Behav. Res. Methods* 39 384–389. 10.3758/bf03193007 17958149 PMC2819369

[B28] Marsh H. W. (1986). Verbal and math self-concepts: an internal/external frame of reference model. *Am. Educ. Res. J.* 23 129–149. 10.3102/00028312023001129

[B29] Marsh H. W. Lüdtke O. Nagengast B. Trautwein U. Abduljabbar A. S. Abdelfattah F. (2015). Dimensional comparison theory: paradoxical relations between self-beliefs and achievements in multiple domains. *Learn. Instruct.* 35 16–32. 10.1016/j.learninstruc.2014.08.005

[B30] Marsh H. W. Pekrun R. Parker P. D. Murayama K. Guo J. Dicke T. (2018). The murky distinction between self-concept and self-efficacy: beware of lurking jingle-jangle fallacies. *J. Educ. Psychol.* 111 331–353. 10.1037/edu0000281

[B31] Marsh H. W. Trautwein U. Lüdtke O. Köller O. Baumert J. (2005). Academic self-concept, interest, grades, and standardized test scores: reciprocal effects models of causal ordering. *Child Dev.* 76 397–416. 10.1111/j.1467-8624.2005.00853.x 15784090

[B32] Marsh H. W. Yeung A. S. (1998). Longitudinal structural equation models of academic self-concept and achievement: gender differences in the development of math and English constructs. *Am. Educ. Res. J.* 35 705–738. 10.3102/00028312035004705

[B33] Meece J. L. Wigfield A. Eccles J. S. (1990). Predictors of math anxiety and its influence on young adolescents’ course enrollment intentions and performance in mathematics. *J. Educ. Psychol.* 82 60–70. 10.1037/0022-0663.82.1.60

[B34] Möller J. Köller O. (2001). Dimensional comparisons: an experimental approach to the internal/external frame of reference model. *J. Educ. Psychol.* 93:826 10.1037//0022-0663.93.4.826

[B35] Möller J. Marsh H. W. (2013). Dimensional comparison theory. *Psychol. Rev.* 120 544–560. 10.1037/a0032459 23544443

[B36] Möller J. Pohlmann B. Köller O. Marsh H. W. (2009). A meta-analytic path analysis of the internal/external frame of reference model of academic achievement and academic self-concept. *Rev. Educ. Res.* 79 1129–1167. 10.3102/0034654309337522

[B37] Möller J. Retelsdorf J. Köller O. Marsh H. W. (2011). The reciprocal internal/external frame of reference model: an integration of models of relations between academic achievement and self-concept. *Am. Educ. Res. J.* 48 1315–1346. 10.1037/dev0000393 29172566

[B38] Muthén L. Muthén B. (1998–2019). *Mplus User’s Guide.* Los Angeles, CA: Muthén & Muthén.

[B40] Nagy G. Trautwein U. Baumert J. Köller O. Garrett J. (2006). Gender and course selection in upper secondary education: effects of academic self-concept and intrinsic value. *Educ. Res. Eval.* 12 323–345. 10.1080/13803610600765687

[B41] National Center for O*Net Development (2014). *O^∗^NET OnLine*. Available at: http://www.onetonline.org/ (accessed July 17 2014).

[B42] Niepel C. Brunner M. Preckel F. (2014). The longitudinal interplay of students’ academic self-concepts and achievements within and across domains: replicating and extending the reciprocal internal/external frame of reference model. *J. Educ. Psychol.* 106 1170–1191. 10.1037/a0036307

[B43] Parker P. D. Schoon I. Tsai Y.-M. Nagy G. Trautwein U. Eccles J. S. (2012). Achievement, agency, gender, and socioeconomic background as predictors of postschool choices: a multicontext study. *Dev. Psychol.* 48 1629–1642. 10.1037/a0029167 22799584

[B44] Schneider B. Stevenson D. (1999). The ambitious generation. *Educ. Leadersh.* 57 22–25.

[B45] Steele C. M. (1997). A threat in the air: how stereotypes shape intellectual identity and performance. *Am. Psychol.* 52 613–629. 10.1037//0003-066x.52.6.613 9174398

[B46] Steinmayr R. Spinath B. (2010). Konstruktion und erste Validierung einer Skala zur Erfassung subjektiver schulischer Werte (SESSW) [construction and validation of a scale for the assessment of subjective task values (SESSW)]. *Diagnostica* 56 195–211. 10.1026/0012-1924/a000023

[B47] Su R. Rounds J. (2015). All STEM fields are not created equal: people and things interests explain gender disparities across STEM fields. *Front. Psychol.* 6:189. 10.3389/fpsyg.2015.00189 25762964 PMC4340183

[B48] Tanaka J. S. (1993). “Multifaceted conceptions of fit in structural equation models,” in *Testing Structural Equation Models* eds Bollen K. A. Long J. S. (Newbury Park, CA: Sage) 10–39.

[B49] Tenenbaum H. R. Leaper C. (2003). Parent-child conversations about science: the socialization of gender inequities? *Dev. Psychol.* 39 34–47. 10.1037/0012-1649.39.1.34 12518807

[B50] Tiedemann J. (2002). Teachers’ gender stereotypes as determinants of teacher perceptions in elementary school mathematics. *Educ. Stud. Math.* 50 49–62.

[B51] Wang M.-T. (2012). Educational and career interests in math: a longitudinal examination of the links between classroom environment, motivational beliefs, and interests. *Dev. Psychol.* 48 1643–1657. 10.1037/a0027247 22390667

[B52] Wang M.-T. Degol J. L. (2017). Gender gap in science, technology, engineering, and mathematics (STEM): current knowledge, implications for practice, policy, and future directions. *Educ. Psychol. Rev.* 29 119–140. 10.1007/s10648-015-9355-x 28458499 PMC5404748

[B53] Wang M.-T. Eccles J. Kenny S. (2013). Not lack of ability but more choice individual and gender differences in choice of careers in science, technology, engineering, and mathematics. *Psychol. Sci.* 24 770–775. 10.1177/0956797612458937 23508740

[B54] Watson J. McEwen A. Dawson S. (1994). Sixth form A level students’ perceptions of the difficulty, intellectual freedom, social benefit and interest of science and arts subjects. *Res. Sci. Technol. Educ.* 12 43–52. 10.1080/0263514940120106

[B55] Watt H. M. G. (2004). Development of adolescents’ self-perceptions, values, and task perceptions according to gender and domain in 7th-through 11th-grade Australian students. *Child Dev.* 75 1556–1574. 10.1111/j.1467-8624.2004.00757.x 15369531

[B56] Watt H. M. G. (2016). “Gender and motivation,” in *Handbook of Motivation at School* eds Wentzel K. Miele D. (New York, NY: Routledge) 320–339.

[B57] Watt H. M. G. Shapka J. D. Morris Z. A. Durik A. M. Keating D. P. Eccles J. S. (2012). Gendered motivational processes affecting high school mathematics participation, educational aspirations, and career plans: a comparison of samples from Australia, Canada, and the United States. *Dev. Psychol.* 48 1594–1611. 10.1037/a0027838 22468566

[B58] Wigfield A. Eccles J. S. (2000). Expectancy–value theory of achievement motivation. *Contemp. Educ. Psychol.* 25 68–81. 10.1006/ceps1999.1015 10620382

[B59] Williams J. MacKinnon D. P. (2008). Resampling and distribution of the product methods for testing indirect effects in complex models. *Struct. Equ. Model.* 15 23–51. 10.1080/10705510701758166 20179778 PMC2825896

[B60] Wolter I. Braun E. Hannover B. (2015). Reading is for girls!? the negative impact of preschool teachers’ traditional gender role attitudes on boys’ reading related motivation and skills. *Front. Psychol.* 6:1267. 10.3389/fpsyg.2015.01267 26379592 PMC4547006

[B61] Yeung A. S. Lau S. Nie Y. (2011). Primary and secondary students’ motivation in learning english: grade and gender differences. *Contemp. Educ. Psychol.* 36 246–256. 10.1016/j.cedpsych.2011.03.001

