# Supplementary material for: Gendered Paths Into STEM-Related and Language-Related Careers: Girls’ and Boys’ Motivational Beliefs and Career Plans in Math and Language Arts
Source: Front Psychol. 2019 Jun 6;10:1243. doi: 10.3389/fpsyg.2019.01243 (PMC6563766; doi:10.3389/fpsyg.2019.01243)
Supplement: Supplementary file 1 [file Data_Sheet_1.docx]

**Appendix A**

*Wording of the Items used in the Analyses*

| Academic self-concept | |
| --- | --- |
| Introductory sentence: ‘In your opinion, which of the following statements applies to you?’ | |
| 1 | I think for [math/language arts] I am…  1 - not at all talented  2 - not talented  3 - moderately talented  4 - talented  5 - very talented |
| 2 | I manage tasks in [math/language arts]….  1 - worse than in the past  2 - mostly worse than in the past  3 - equally well compared to the past  4 - mostly better than in the past  5 - better than in the past |
| 3 | I think for [mathematics/language-arts] I am…  1 - less talented than my classmates  2 - mostly less talented than my classmates  3 - equally talented compared to my classmates  4 - mostly more talented than my classmates  5 - more talented than my classmates |
| 6 | When I look at what I should be able to do in mathematics, I think I am…  1 - not at all talented  2 - rather not talented  3 - moderately talented  4 - rather talented  5 - very talented |
| Intrinsic value | |
| Introductory sentence: ‘To what extent do the following statements apply to you?’ | |
| 1 | I like doing [mathematics/language arts]. |
| 2 | [Mathematics/language arts] is fun to me. |
| 3 | [Mathematics/language arts] is interesting. |
| Utility value | |
| Introductory sentence:  ‘To what extent do the following statements apply to you?’ | |
| 1 | I find that the things that I learn in [mathematics/language arts] are generally important for my life. |
| 2 | [Mathematics/language-arts] is important für my future. |
| 3 | The content that I learn in [mathematics/language-arts] will help me in my life. |
| Career plans | |
| Open answer format | What do you think, which occupation will you have when you are 30years old? |

**Appendix B**

Table 1

*Measurement Invariance Test Across Gender Groups for Mathematics*

| **Time 1** | | | | **Time 2** | | | |
| --- | --- | --- | --- | --- | --- | --- | --- |
| **Intrinsic value** | | | | | | | |
|  | step1 | step2 | step3 |  | step1 | step2 | step3 |
| χ² | 0.000 | 5.673 | 11.954 | χ² | 0.000 | 4.577 | 5.125 |
| *df* | 0 | 2 | 4 | *df* | 0 | 2 | 4 |
| CFI | 1.000 | 0.998 | 0.996 | CFI | 1.000 | 0.997 | 0.999 |
| RMSEA | 0.000 | 0.058 | 0.06 | RMSEA | 0.000 | 0.059 | 0.028 |
| SRMR | 0.000 | 0.037 | 0.052 | SRMR | 0.000 | 0.03 | 0.031 |
| Δ CFI |  | -0.002 | -0.002 | Δ CFI |  | -0.003 | 0.002 |
| Δ RMSEA |  | 0.058 | 0.002 | Δ RMSEA |  | 0.059 | -0.031 |
| Δ SRMR |  | 0.037 | 0.015 | Δ SRMR |  | 0.030 | 0.001 |
| **Utility value** | | | | | | | |
| χ² | 0.000 | 1.037 | 1.88 | χ² | 0.000 | 0.335 | 1.329 |
| *df* | 0 | 2 | 4 | *df* | 0 | 2 | 4 |
| CFI | 1.000 | 1.000 | 1.000 | CFI | 1.000 | 1.000 | 1.000 |
| RMSEA | 0.000 | 0.000 | 0.000 | RMSEA | 0.000 | 0.000 | 0.000 |
| SRMR | 0.000 | 0.013 | 0.018 | SRMR | 0.000 | 0.008 | 0.008 |
| Δ CFI |  | 0.000 | 0.000 | Δ CFI |  | 0.000 | 0.000 |
| Δ RMSEA |  | 0.000 | 0.000 | Δ RMSEA |  | 0.000 | 0.000 |
| Δ SRMR |  | 0.013 | 0.005 | Δ SRMR |  | 0.008 | 0.000 |
| **Academic self-concept** | | | | | | | |
| χ² | 4.061 | 6.346 | 19.277 | χ² | 0.000 | 6.750 | 14.302 |
| *df* | 4 | 7 | 10 | *df* | 4 | 7 | 10 |
| CFI | 1.000 | 1.000 | 0.994 | CFI | 1.000 | 1.000 | 0.996 |
| RMSEA | 0.005 | 0.000 | 0.041 | RMSEA | 0.000 | 0.000 | 0.034 |
| SRMR | 0.007 | 0.020 | 0.031 | SRMR | 0.002 | 0.045 | 0.046 |
| Δ CFI |  | 0.000 | -0.006 | Δ CFI |  | 0 | -0.004 |
| Δ RMSEA |  | -0.005 | 0.041 | Δ RMSEA |  | 0 | 0.034 |
| Δ SRMR |  | 0.013 | 0.011 | Δ SRMR |  | 0.043 | 0.001 |

*Note.* step 1 = factor loadings and item intercepts variant across groups; step 2 = item intercepts variant, factor loadings constrained to group-invariance; step 3 = factor loadings and item intercepts group invariant.

Table 2

*Measurement Invariance Test Across Gender Groups for Language-Arts*

| **Time 1** | | | | **Time 2** | | | |
| --- | --- | --- | --- | --- | --- | --- | --- |
| **Intrinsic value** | | | | | | | |
|  | step1 | step2 | step3 |  | step1 | step2 | step3 |
| χ² | 0.000 | 0.598 | 0.885 | χ² | 0.000 | 4.577 | 5 |
| *df* | 0 | 2 | 4 | *df* | 0 | 2 | 4 |
| CFI | 1.000 | 1.000 | 1.000 | CFI | 1.000 | 0.997 | 0.999 |
| RMSEA | 0.000 | 0.000 | 0.000 | RMSEA | 0.000 | 0.059 | 0.028 |
| SRMR | 0.000 | 0.009 | 0.009 | SRMR | 0.000 | 0.030 | 0.031 |
| Δ CFI |  | 0.000 | 0.000 | Δ CFI |  | -0.003 | 0.002 |
| Δ RMSEA |  | 0.000 | 0.000 | Δ RMSEA |  | 0.059 | -0.031 |
| Δ SRMR |  | 0.009 | 0.000 | Δ SRMR |  | 0.030 | 0.001 |
| **Utility value** | | | | | | | |
| χ² | 0.000 | 2.274 | 5.669 | χ² | 0.000 | 1.006 | 3.820 |
| *df* | 0 | 2 | 4 | *df* | 0 | 2 | 4 |
| CFI | 1.000 | 1.000 | 0.998 | CFI | 1.000 | 1.000 | 1.000 |
| RMSEA | 0.000 | 0.016 | 0.028 | RMSEA | 0.000 | 0.000 | 0.000 |
| SRMR | 0.000 | 0.028 | 0.036 | SRMR | 0.000 | 0.021 | 0.025 |
| Δ CFI |  | 0.000 | -0.002 | Δ CFI |  | 0.000 | 0.000 |
| Δ RMSEA |  | 0.016 | 0.012 | Δ RMSEA |  | 0.000 | 0.000 |
| Δ SRMR |  | 0.028 | 0.008 | Δ SRMR |  | 0.021 | 0.004 |
| **Academic self-concept** | | | | | | | |
| χ² | 5.256 | 9.492 | 11.195 | χ² | 18.365 | 23.534 | 35.640 |
| *df* | 4 | 7 | 10 | *df* | 4 | 7 | 10 |
| CFI | 0.99 | 0.999 | 0.999 | CFI | 0.989 | 0.987 | 0.980 |
| RMSEA | 0.024 | 0.026 | 0.015 | RMSEA | 0.099 | 0.08 | 0.083 |
| SRMR | 0.009 | 0.034 | 0.037 | SRMR | 0.019 | 0.046 | 0.044 |
| Δ CFI |  | 0.009 | 0.000 | Δ CFI |  | -0.002 | -0.007 |
| Δ RMSEA |  | 0.002 | -0.011 | Δ RMSEA |  | -0.019 | 0.003 |
| Δ SRMR |  | 0.025 | 0.003 | Δ SRMR |  | 0.027 | -0.002 |

*Note*. step 1 = factor loadings and item intercepts variant across groups; step 2 = item intercepts variant, factor loadings constrained to group-invariance; step 3 = factor loadings and item intercepts group invariant.

Table 3

*Time Invariance Test – Constructs for Math*

| **Intrinsic value  Utility value** | | | | | | | |
| --- | --- | --- | --- | --- | --- | --- | --- |
|  | step1 | step2 | step3 |  | step1 | step2 | step3 |
| χ² | 76.405 | 87.515 | 91.348 | χ² | 62.755 | 70.503 | 82.445 |
| *df* | 8 | 10 | 13 | *df* | 8 | 10 | 13 |
| CFI | 0.979 | 0.977 | 0.976 | CFI | 0.982 | 0.980 | 0.977 |
| RMSEA | 0.088 | 0.083 | 0.074 | RMSEA | 0.078 | 0.074 | 0.069 |
| SRMR | 0.025 | 0.027 | 0.028 | SRMR | 0.023 | 0.026 | 0.035 |
| Δ CFI |  | -0.002 | -0.001 | Δ CFI |  | -0.002 | -0.002 |
| Δ RMSEA |  | -0.005 | -0.009 | Δ RMSEA |  | -0.004 | -0.009 |
| Δ SRMR |  | 0.002 | 0.001 | Δ SRMR |  | 0.003 | 0.012 |
| **Academic self-concept** | | | | | | | |
| χ² | 158.903 | 166.535 | 175.138 |  |  |  |  |
| *df* | 19 | 22 | 26 |  |  |  |  |
| CFI | 0.959 | 0.957 | 0.956 |  |  |  |  |
| RMSEA | 0.081 | 0.077 | 0.072 |  |  |  |  |
| SRMR | 0.034 | 0.035 | 0.039 |  |  |  |  |
| Δ CFI |  | -0.002 | -0.001 |  |  |  |  |
| Δ RMSEA |  | -0.004 | -0.005 |  |  |  |  |
| Δ SRMR |  | 0.001 | 0.004 |  |  |  |  |

Table 4

*Time Invariance Test – Constructs for Language-Arts*

| **Intrinsic value  Utility value** | | | | | | | |
| --- | --- | --- | --- | --- | --- | --- | --- |
|  | step1 | step2 | step3 |  | step1 | step2 | step3 |
| χ² | 32.124 | 36.658 | 40.662 | χ² | 40.566 | 44.760 | 47.225 |
| *df* | 8 | 10 | 13 | *df* | 8 | 10 | 13 |
| CFI | 0.995 | 0.995 | 0.99 | CFI | 0.989 | 0.989 | 0.989 |
| RMSEA | 0.052 | 0.049 | 0.044 | RMSEA | 0.61 | 0.056 | 0.049 |
| SRMR | 0.027 | 0.028 | 0.03 | SRMR | 0.016 | 0.028 | 0.031 |
| Δ CFI |  | 0 | -0.005 | Δ CFI |  | 0 | 0 |
| Δ RMSEA |  | -0.003 | -0.005 | Δ RMSEA |  | -0.554 | -0.007 |
| Δ SRMR |  | 0.001 | 0.002 | Δ SRMR |  | 0.012 | 0.003 |
| **Academic self-concept** | | | | | | | |
| χ² | 153.167 | 157.474 | 173.903 |  |  |  |  |
| *df* | 19 | 22 | 26 |  |  |  |  |
| CFI | 0.966 | 0.966 | 0.963 |  |  |  |  |
| RMSEA | 0.080 | 0.074 | 0.072 |  |  |  |  |
| SRMR | 0.032 | 0.034 | 0.039 |  |  |  |  |
| Δ CFI |  | 0.000 | -0.003 |  |  |  |  |
| Δ RMSEA |  | -0.010 | 0.002 |  |  |  |  |
| Δ SRMR |  | -0.002 | 0.009 |  |  |  |  |

**Appendix C**

Table 1

*Missing Values on the Study Variables at the Student Level and Attrition across Waves*

| Variable | Missings on the student level T1 | Missings on the student level T2 | Attrition rate across waves^1^ |
| --- | --- | --- | --- |
| Self-reported mathematics grade | 1.5% | -- | -- |
| Self-reported grade in language arts | 1.6% | -- | -- |
| Self-concept in mathematics | 0.7% | 32.9% | 32.6% |
| Utility value in mathematics | 0.7% | 32.9% | 32.6% |
| Intrinsic value in mathematics | 0.7% | 32.9% | 32.6% |
| Self-concept in language arts | 0.4% | 32.9% | 32.6% |
| Utility value in language arts | 1.0% | 32.9% | 32.6% |
| Intrinsic value in language arts | 1.0% | 32.9% | 32.6% |
| Career plans related to mathematics | 31.4% | 54.3% | 47.1% |
| Career plans related to language arts | 31.4% | 54.3% | 52.9% |

*Note*. The attrition rate across waves was computed by selecting cases with no missing values at Time 1 and identifying the rate of missing values at Time 2 for only those cases.

Table 2

*Number of Students and Classrooms per Participating School*

| School number | Number of students | Number of classrooms |
| --- | --- | --- |
| 1 | 2 | 52 |
| 2 | 3 | 70 |
| 3 | 5 | 114 |
| 4 | 3 | 74 |
| 5 | 9 | 110 |
| 6 | 2 | 29 |
| 7 | 3 | 63 |
| 8 | 8 | 150 |
| 9 | 4 | 65 |
| 10 | 3 | 56 |
| 11 | 2 | 44 |
| 12 | 8 | 212 |
| 13 | 6 | 78 |

**Appendix D**

Table 1

*Relations between Academic Self-concept, Utility Value, Intrinsic Value and Career Plans: Self-Concept*

| Variable | Self-concept math T1 | | | Self-concept lang T1 | | | Self-concept math T2 | | | Self-concept lang T2 | | |
| --- | --- | --- | --- | --- | --- | --- | --- | --- | --- | --- | --- | --- |
|  | *β* | *SE* | *p* | *β* | *SE* | *p* | *β* | *SE* | *p* | *β* | *SE* | *p* |
| Girls | **-0.22** | **0.03** | **<.001** | **-0.07** | **0.03** | **.006** | *-0.04* | *0.03* | *.146* | 0.04 | 0.03 | .121 |
| German native | **-0.09** | **0.03** | **.001** | 0.01 | 0.03 | .917 | 0.05 | 0.03 | .083 | 0.01 | 0.03 | .690 |
| Math achiev | **0.69** | **0.03** | **<.001** | **-0.38** | **0.04** | **<.001** | *0.06* | *0.04* | *.119* | -0.02 | 0.05 | .700 |
| Lang achiev | -0.05 | 0.04 | .195 | **0.65** | **0.04** | **<.001** | 0.04 | 0.04 | .296 | 0.09 | 0.05 | .064 |
| Comp. school | -0.01 | 0.03 | .934 | 0.04 | 0.04 | .381 | 0.02 | 0.03 | .385 | -0.01 | 0.03 | .906 |
| Career math T1 |  |  |  |  |  |  | 0.02 | 0.03 | .432 | -0.03 | 0.03 | .295 |
| Career lang T1 |  |  |  |  |  |  | -0.01 | 0.02 | .968 | 0.04 | 0.02 | .121 |
| Self math T1 |  |  |  |  |  |  | **0.70** | **0.06** | **<.001** | -0.03 | 0.08 | .715 |
| Self lang T1 |  |  |  |  |  |  | 0.01 | 0.04 | .890 | **0.53** | **0.07** | **<.001** |
| Utility math T1 |  |  |  |  |  |  | 0.04 | 0.03 | .139 | 0.05 | 0.04 | .241 |
| Utility lang T1 |  |  |  |  |  |  | -0.01 | 0.03 | .722 | 0.01 | 0.04 | .728 |
| Intrinsic math T1 |  |  |  |  |  |  | 0.02 | 0.07 | .732 | -0.04 | 0.07 | .572 |
| Intrinsic lang T1 |  |  |  |  |  |  | -0.06 | 0.04 | .183 | ***0.16*** | ***0.06*** | ***0.01*** |

*Note*. N = 1117; German native = German native language; Math achiev = Self-reported grade Mathematics (recoded); Lang achiev = Self-reported grade German (recoded); Comp school = Comprehensive school (‘Integrierte Sekundarschule’); Career math T1/T2 = career plans in math-related fields at Time 1/ Time 2; Career lang T1/T2 = career plans in language domain at Time 1/ Time 2; Self math T1= Self-concept in mathematics at Time 1; Self lang T1= self-concept in language arts at Time 1.

Model fit: χ² (947) = 2189.099, CFI = 0.957, TLI = 0.947, RMSEA = 0.034, SRMR = 0.072.

| Variable | Utility math T1 | | | Utility lang T1 | | | Utility math T2 | | | Utility lang T2 | | |
| --- | --- | --- | --- | --- | --- | --- | --- | --- | --- | --- | --- | --- |
|  | *β* | *SE* | *p* | *β* | *SE* | *p* | *β* | *SE* | *p* | *β* | *SE* | *p* |
| Girls | **-0.13** | **0.04** | **<.001** | 0.06 | 0.04 | .169 | -0.03 | 0.04 | .517 | 0.04 | 0.03 | .121 |
| German native | **-0.13** | **0.04** | **.001** | ***-0.08*** | ***0.04*** | ***.041*** | ***0.12*** | ***0.03*** | ***<.001*** | 0.01 | 0.03 | .690 |
| Math achiev | **0.24** | **0.04** | **<.001** | **-0.10** | **0.04** | **<.001** | 0.04 | 0.05 | .453 | -0.02 | 0.05 | .700 |
| Lang achiev | -0.02 | 0.04 | .733 | **0.25** | **0.04** | **<.001** | 0.07 | 0.05 | .139 | 0.09 | 0.05 | .064 |
| Comp. school | ***0.17*** | ***0.05*** | ***<.001*** | ***0.15*** | ***0.04*** | ***.001*** | 0.07 | 0.04 | .064 | -0.01 | 0.03 | .906 |
| Career math T1 |  |  |  |  |  |  | -0.02 | 0.04 | .564 | -0.03 | 0.03 | .295 |
| Career lang T1 |  |  |  |  |  |  | -0.03 | 0.03 | .182 | 0.04 | 0.02 | .121 |
| Self math T1 |  |  |  |  |  |  | 0.06 | 0.09 | .471 | -0.16 | 0.09 | .080 |
| Self lang T1 |  |  |  |  |  |  | ***-0.10*** | ***0.04*** | ***.019*** | 0.06 | 0.05 | .252 |
| Utility math T1 |  |  |  |  |  |  | **0.54** | **0.05** | **<.001** | 0.03 | 0.04 | .513 |
| Utility lang T1 |  |  |  |  |  |  | 0.02 | 0.04 | .633 | **0.48** | **0.40** | **<.001** |
| Intrinsic math T1 |  |  |  |  |  |  | 0.01 | 0.09 | .905 | 0.10 | 0.07 | .153 |
| Intrinsic lang T2 |  |  |  |  |  |  | -0.03 | 0.05 | .577 | ***0.10*** | ***0.05*** | ***.036*** |

Table 2

*Relations between Academic Self-concept, Utility Value, Intrinsic Value and Career Plans: Utility Value*

*Note*. N = 1117; German native = German native language; Math achiev = Self-reported grade Mathematics (recoded); Lang achiev = Self-reported grade German (recoded); Comp school = Comprehensive school (‘Integrierte Sekundarschule’); Career math T1/T2 = career plans in math-related fields at Time 1/ Time 2; Career lang T1/T2 = career plans in language domain at Time 1/ Time 2; Self math T1= Self-concept in mathematics at Time 1; Self lang T1= self-concept in language arts at Time 1.

Model fit: χ² (947) = 2189.099, CFI = 0.957, TLI = 0.947, RMSEA = 0.034, SRMR = 0.072.

Table 3

| Variable | Intrinsic math T1 | | | Intrinsic lang T1 | | | Intrinsic math T2 | | | Intrinsic lang T2 | | |
| --- | --- | --- | --- | --- | --- | --- | --- | --- | --- | --- | --- | --- |
|  | *β* | *SE* | *p* | *β* | *SE* | *p* | *β* | *SE* | *p* | *β* | *SE* | *p* |
| Girls | **-0.18** | **0.03** | **<.001** | 0.04 | 0.03 | .139 | 0.01 | 0.03 | .741 | 0.02 | 0.03 | .486 |
| German native | *-0.06* | *0.03* | *.091* | -0.01 | 0.03 | .823 | 0.04 | 0.02 | .116 | 0.02 | 0.04 | .594 |
| Math achiev | **0.53** | **0.04** | **<.001** | **-0.36** | **0.03** | **<.001** | 0.06 | 0.05 | .200 | 0.01 | 0.05 | .790 |
| Lang achiev | ***-0.10*** | ***0.04*** | ***.016*** | **0.48** | **0.04** | **<.001** | 0.04 | 0.05 | .451 | 0.07 | 0.05 | .132 |
| Comp. school | 0.01 | 0.04 | .957 | 0.06 | 0.05 | .234 | -0.01 | 0.03 | .873 | -0.01 | 0.03 | .790 |
| Career math T1 |  |  |  |  |  |  | 0.02 | 0.03 | .510 | -0.04 | 0.04 | .310 |
| Career lang T1 |  |  |  |  |  |  | -0.04 | 0.02 | .074 | 0.03 | 0.03 | .206 |
| Self math T1 |  |  |  |  |  |  | ***0.21*** | ***0.08*** | ***.010*** | ***-0.18*** | ***0.07*** | ***.010*** |
| Self lang T1 |  |  |  |  |  |  | ***-0.13*** | ***0.04*** | ***.003*** | 0.10 | 0.06 | .112 |
| Utility math T1 |  |  |  |  |  |  | 0.03 | 0.04 | .379 | 0.07 | 0.04 | 0.08 |
| Utility lang T1 |  |  |  |  |  |  | 0.04 | 0.03 | .153 | 0.01 | 0.03 | .922 |
| Intrinsic math T1 |  |  |  |  |  |  | **0.48** | **0.07** | **<.001** | 0.04 | 0.05 | .388 |
| Intrinsic lang T1 |  |  |  |  |  |  | 0.05 | 0.05 | .304 | **0.60** | **0.05** | **<.001** |

*Relations between Academic Self-concept, Utility Value, Intrinsic Value and Career Plans: Intrinsic Value*

*Note*. N = 1117; German native = German native language; Math achiev = Self-reported grade Mathematics (recoded); Lang achiev = Self-reported grade German (recoded); Comp school = Comprehensive school (‘Integrierte Sekundarschule’); Career math T1/T2 = career plans in math-related fields at Time 1/ Time 2; Career lang T1/T2 = career plans in language domain at Time 1/ Time 2; Self math T1= Self-concept in mathematics at Time 1; Self lang T1= self-concept in language arts at Time 1.

Model fit: χ² (947) = 2189.099, CFI = 0.957, TLI = 0.947, RMSEA = 0.034, SRMR = 0.072.

|  | Career math T1 | | | Career lang T1 | | | Career math T2 | | | | Career lang T2 | | |
| --- | --- | --- | --- | --- | --- | --- | --- | --- | --- | --- | --- | --- | --- |
|  | *β* | *SE* | *p* | *β* | *SE* | *p* | *β* | | *SE* | *p* | *β* | *SE* | *p* |
| Girls | **-0.11** | **0.04** | **.002** | **0.10** | **0.04** | **.009** | -0.05 | | 0.03 | .129 | 0.07 | 0.04 | .106 |
| German native | -0.06 | 0.04 | .117 | **-0.06** | **0.03** | **.033** | -0.02 | | 0.04 | .552 | 0.04 | 0.04 | .247 |
| Math achiev | **0.17** | **0.05** | **<.001** | -0.05 | 0.04 | .226 | -0.08 | | 0.06 | .139 | 0.02 | 0.06 | .732 |
| Lang achiev | -0.02 | 0.03 | .537 | **0.12** | **0.05** | **.008** | 0.06 | | 0.05 | .260 | -0.03 | 0.05 | .608 |
| Comp school | **-0.23** | **0.03** | **<.001** | **-0.22** | **0.03** | **<.001** | -0.09 | | 0.05 | .088 | **-0.14** | **0.04** | **.005** |
| Self math T1 |  |  |  |  |  |  | 0.14 | 0.08 | | .077 | 0.07 | 0.10 | .515 |
| Self lang T1 |  |  |  |  |  |  | -0.01 | 0.05 | | .943 | 0.01 | 0.06 | .971 |
| Utility math T1 |  |  |  |  |  |  | 0.02 | 0.05 | | .726 | 0.05 | 0.05 | .344 |
| Utility lang T1 |  |  |  |  |  |  | -0.03 | 0.04 | | .539 | ***0.11*** | ***0.05*** | ***.035*** |
| Intrinsic math T1 |  |  |  |  |  |  | -0.01 | 0.06 | | .831 | -0.08 | 0.09 | .347 |
| Intrinsic lang T1 |  |  |  |  |  |  | -0.04 | 0.06 | | .520 | -0.06 | 0.06 | .341 |
| Career math T1 |  |  |  |  |  |  | **0.56** | **0.05** | | **<.001** | -0.01 | 0.04 | .861 |
| Career lang T1 |  |  |  |  |  |  | -0.07 | 0.04 | | .144 | **0.58** | **0.06** | **<.001** |
| *Note.* *N* = 1117; German native = German native language; Math achiev = Self-reported grade Mathematics (recoded); Lang achiev = Self-reported grade German (recoded); Comp school = Comprehensive school (‘Integrierte Sekundarschule’); Career math T1/T2 = career plans in math-related fields at Time 1/ Time 2; Career lang T1/T2 = career plans in language domain at Time 1/ Time 2; Self math T1= Self-concept in mathematics at Time 1; Self lang T1= self-concept in language arts at Time 1.  The model fit is χ² (947) = 2189.099, CFI = 0.957, TLI = 0.947, RMSEA = 0.034, SRMR = 0.072. | | | | | | | | | | | | | |

Table 4

*Relations between Academic Self-concept, Utility Value, Intrinsic Value and Career Plans: Career Plans*
